# F429 Regulation of Tunnels in Cytochrome P450 2B4: A Top Down Study of Multiple Molecular Dynamics Simulations

**DOI:** 10.1371/journal.pone.0137075

**Published:** 2015-09-28

**Authors:** Giordano Mancini, Costantino Zazza

**Affiliations:** 1 Scuola Normale Superiore di Pisa, Piazza dei Cavalieri 7, 56126, Pisa, Italy, and Istituto Nazionale di Fisica Nucleare (INFN) sezione di Pisa, Largo Bruno Pontecorvo 3, 56127, Pisa, Italy; 2 Università degli Studi di Roma “La Sapienza”, Piazzale Aldo Moro 5, 00185, Roma, Italy; Instituto de Tecnologica Química e Biológica, UNL, PORTUGAL

## Abstract

The root causes of the outcomes of the single-site mutation in enzymes remain by and large not well understood. This is the case of the F429H mutant of the cytochrome P450 (CYP) 2B4 enzyme where the substitution, on the proximal surface of the active site, of a conserved phenylalanine 429 residue with histidine seems to hamper the formation of the active species, Compound I (porphyrin cation radical-Fe^(IV)^ = O, Cpd I) from the ferric hydroperoxo (Fe^(III)^OOH^-^, Cpd 0) precursor. Here we report a study based on extensive molecular dynamic (MD) simulations of 4 CYP-2B4 point mutations compared to the WT enzyme, having the goal of better clarifying the importance of the proximal Phe429 residue on CYP 2B4 catalytic properties. To consolidate the huge amount of data coming from five simulations and extract the most distinct structural features of the five species studied we made an extensive use of cluster analysis. The results show that all studied single polymorphisms of F429, with different side chain properties: *i)* drastically alter the reservoir of conformations accessible by the protein, perturbing global dynamics *ii)* expose the thiolate group of residue Cys436 to the solvent, altering the electronic properties of Cpd0 and *iii*) affect the various ingress and egress channels connecting the distal sites with the bulk environment, altering the reversibility of these channels. In particular, it was observed that the wild type enzyme exhibits unique structural features as compared to all mutant species in terms of weak interactions (hydrogen bonds) that generate a completely different dynamical behavior of the complete system. Albeit not conclusive, the current computational investigation sheds some light on the subtle and critical effects that proximal single-site mutations can exert on the functional mechanisms of human microsomal CYPs which should go rather far beyond local structure characterization.

## Introduction

Cytochrome P450s (CYPs) form a ubiquitous enzyme family which is directly involved in the oxidation of a wide range of organic compounds including drugs, steroids, and vitamins.[1] The importance of CYPs in nature is witnessed by their presence in all forms of life, from archea to animalia, including mammals. For instance, in the human genome, there are 57 different genes involved in CYP expression.[2] Due to their activity and diversity,[3–5] CYPs have attracted massive studies designed to understand the factors of their activity.

**Fig 1:**
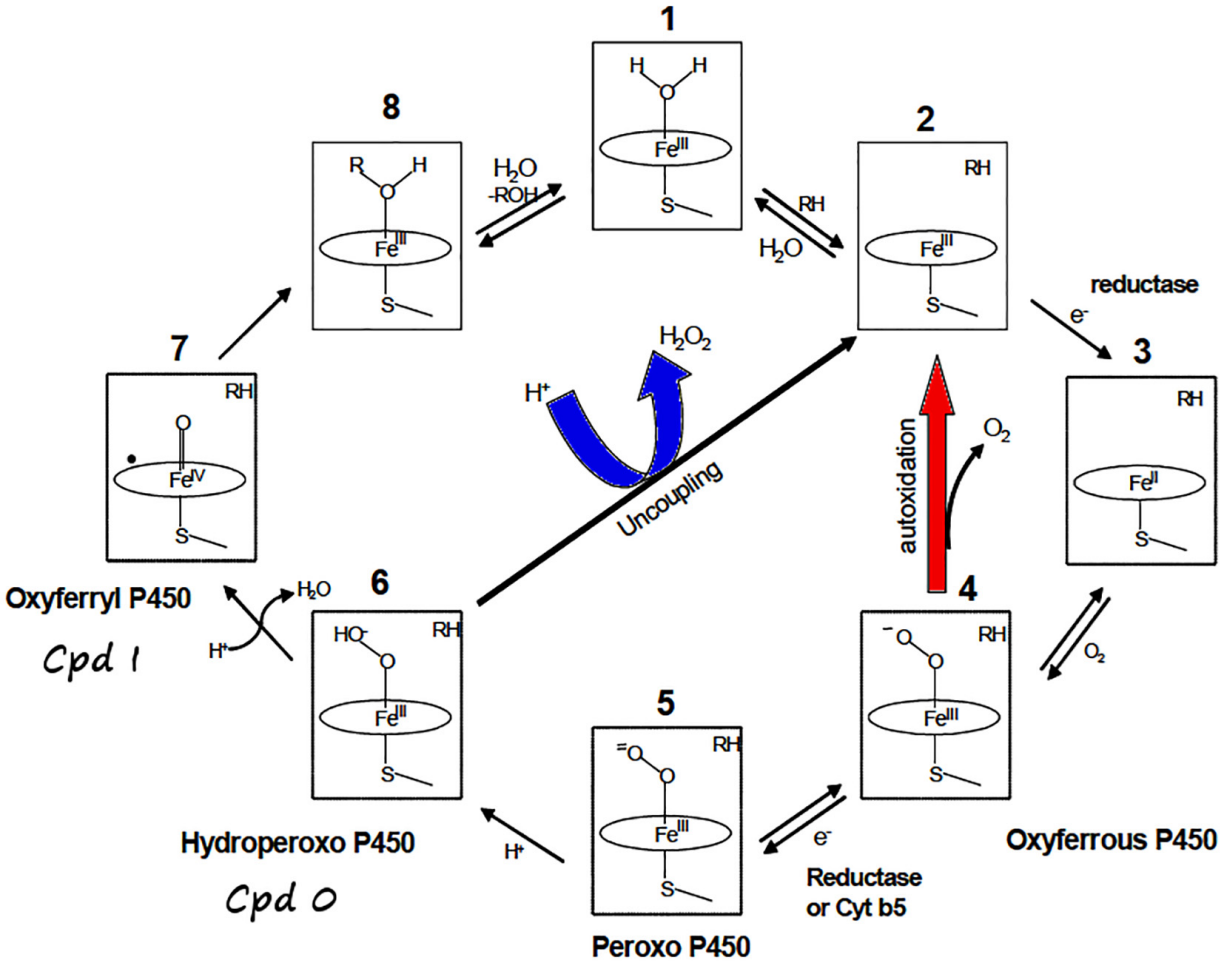
A schematic catalytic cycle for CYPs, showing all the key species, and the uncoupling process.

The active site of CYP contains a heme group buried inside the protein matrix and linked to a conserved cysteine residue with a Fe-thiolate bond.[6,7] This implies that substrates in the bulk solution can interact with the CYPs active site if and only if they are capable of permeating the functional channels connecting the active site with the protein surface which varies between the isoforms.[8–13] In the absence of the substrate, the Fe^(III)^ ion is hexacoordinated with a covalently bound water molecule occupying the sixth-position opposite to the cysteine-thiolate ligand, so-called resting ferric state shown in **1** in Fig 1.[14,15] The entrance of the substrate (RH in Fig 1) to the distal region of the active site causes the displacement of the water molecule bound to the heme ion, to generate **2**. Furthermore, the entrance of the substrate is generally accompanied by the exclusion of all excess water molecules from the vicinity of the heme group, through exit pathways generally different from the substrate access channel.[16] Such a dehydration of the catalytic site is essential for starting the enzymatic cycle of cytochrome P450 proteins,[17] since this favors the reduction of the ferric species **2** by cyt P450 reductase (CPR, first Electron Transfer, ET) due to increase of the Fe^(II)^/Fe^(III)^ redox equilibrium towards the ferrous counterpart **3**.[18–20]. Subsequently, O2 binds to the heme iron (4), which then undergoes a second reduction (5) and a protonation to form the Fe^(III)^OOH^-^ intermediate **6**, called Cpd 0. The latter undergoes in turn, a second protonation on the distal OH, resulting in heterolytic O-OH bond cleavage, and generation of the iron-oxo porphyrin-cation radical intermediate, Por+• Fe^(IV)^ = O **7**, so called Cpd I.[21–26] This Por+• Fe^(IV)^ = O species, which is the ultimate oxidant of the enzyme, catalyzes the efficient mono-oxygenation of many different substrates, e.g., **8**.[14,24,27–33] Alternatively, the protonation of the proximal oxygen atom of the Cpd 0 species is responsible for the release of hydrogen peroxide (H_2_O_2_), thereby uncoupling the oxygen consumption and substrate oxidation (see central arrow in Fig 1).[34–36] The overall coupled process may be then summarized as follows:

$$RH+O_{2}+2e^{-}+2H^{+}\to R-OH+H_{2}O$$

A detailed understanding of the factors modulating the enormous catalytic power of human cytochrome P450s is therefore of crucial importance. This group of enzymes in not only involved in the efficient hydroxylation of important natural and synthetic compounds;[1,5,37–39] they also catalyze the oxidation of environmental pollutants to which the human body may be exposed, making them more soluble for easier elimination.[40] In the course of analyzing the activity of drug metabolizing CYPs, many enzymes were found to be nonspecific and capable of hydroxylating non-native substrates.[1,3–5,40,41] The fact that drug-metabolizing CYPs are not specific is due to their conformational plasticity:[11] these enzymes feature adaptive structures capable of accommodating different reaction partners while essentially maintaining the same fold in all CYPs. This intriguing aspect has been attributed to the presence of secondary structural elements having relatively high degrees of flexibility.[11,12] It has been shown that substrate and water pathways follow different routes and that water access to the active site is regulated by the interaction of residue R375 with the heme propionate groups CYP-3A4[13,16], which is able to switch between different conformations, allowing a fine control over the presence of water inside the pocket.

Previously Davydov et. al.[42] had unequivocally showed that, starting from the same amounts of Cpd 0, the Phe429→His mutant of CYP 2B4 exhibits, in the presence of the butylated hydroxytoluene (BHT) substrate, only 4−5% product formation (e.g. hydroxymethyl BHT and 3-hydroxy-tert-butyl BHT) compared with the wild-type (WT, 2B4) form. In a recent communication, Quantum-Mechanical/ Molecular Mechanical (QM/MM) calculations showed an increased propensity of the F429H mutant enzyme to undergo homolysis of the O-OH bond of Cpd 0, rather than the heterolysis exhibited by the WT enzyme. The root cause of this behavior was shown to be the formation of a hydrogen bond (H-bond), connecting the His429 to the Cys436 in the mutant enzyme, which increased the propensity for bond homolysis.[43] The QM/MM calculations followed molecular dynamics (MD) simulations which established the existence of this H-bond between His429 and the thiolate of the cysteine ligand. The results were later confirmed by a spectroscopic investigation of a newly obtained CYP 2B4 F429H crystal structure.[44]

In this contribution, we tried to push forward the phase space exploration of CYP-2B4 by extensive MD simulations characterizing the WT enzyme and 4 different mutations of the F429 residue, namely F429A, F429E, F429H and F429L for a total of 0.75 μs of sampling time. Target mutations were selected in order to provide a wide range of hydrophilic/hydrophobic character and side chain bulk. This large scale investigation of the global mechanical effects induced by mutations on CYP 2B4 mechanical properties allowed us to further elucidate the role of F429, by searching for shared, frequently visited and statistically relevant, conformational sub spaces and alteration of specific contacts in all the trajectories. Several unique features of the native enzyme as compared to the 4 mutants have been found, even when F429 is mutated into another bulky hydrophobic residue such as Leu. Moreover, the results show clearly that the functional impact of this point mutation are not limited to the modes of O−OH bond cleavage in the WT and F429H enzymes,[43] but are extended to the whole enzyme dynamics, altering inter domain contacts (salt bridges and hydrogen bonds) and access pathways to the active site. Note that F429H has already been the object of a MD investigation[43] and for this reason we have omitted in this study results that have been already discussed previously. Here, our intention was to (*i*) to test whether the mutation of Phe has “long range” effects in addition to the local disruption of the active site electronic properties which further explain the selective pressure that make this residue highly conserved and (*ii*) to further investigate which unique properties of the F429H mutant observed in the experiments were caused by the removal of Phe429 or by the presence of a histidine residue.

The manuscript is organized as follows: after a summary of the methods used to set up, run (section 2.1) and analyze the five trajectories (section 2.2) the whole conformational space sampled in the 750 ns is analyzed in section 3.1 mainly by cluster analysis; an accurate assessment of the frames used to cluster and on the parameters is given in the Supporting Information (section S3.1). Section 3.2 includes covariance and fluctuation analysis of the studied systems. Section 3.3 compares the five trajectories in finer detail using hydrogen bond and salt-bridge statistics and solvent distribution around critical groups (e.g. Cys436) and includes also a closer inspection of the representative structures selected by the collective analysis presented in section 3.1, including solvent pathways. All the results are then discussed together in section 4, while section 5 includes a few concluding remarks.

## Computational Details

### 2.1 System setup

**Fig 2:**
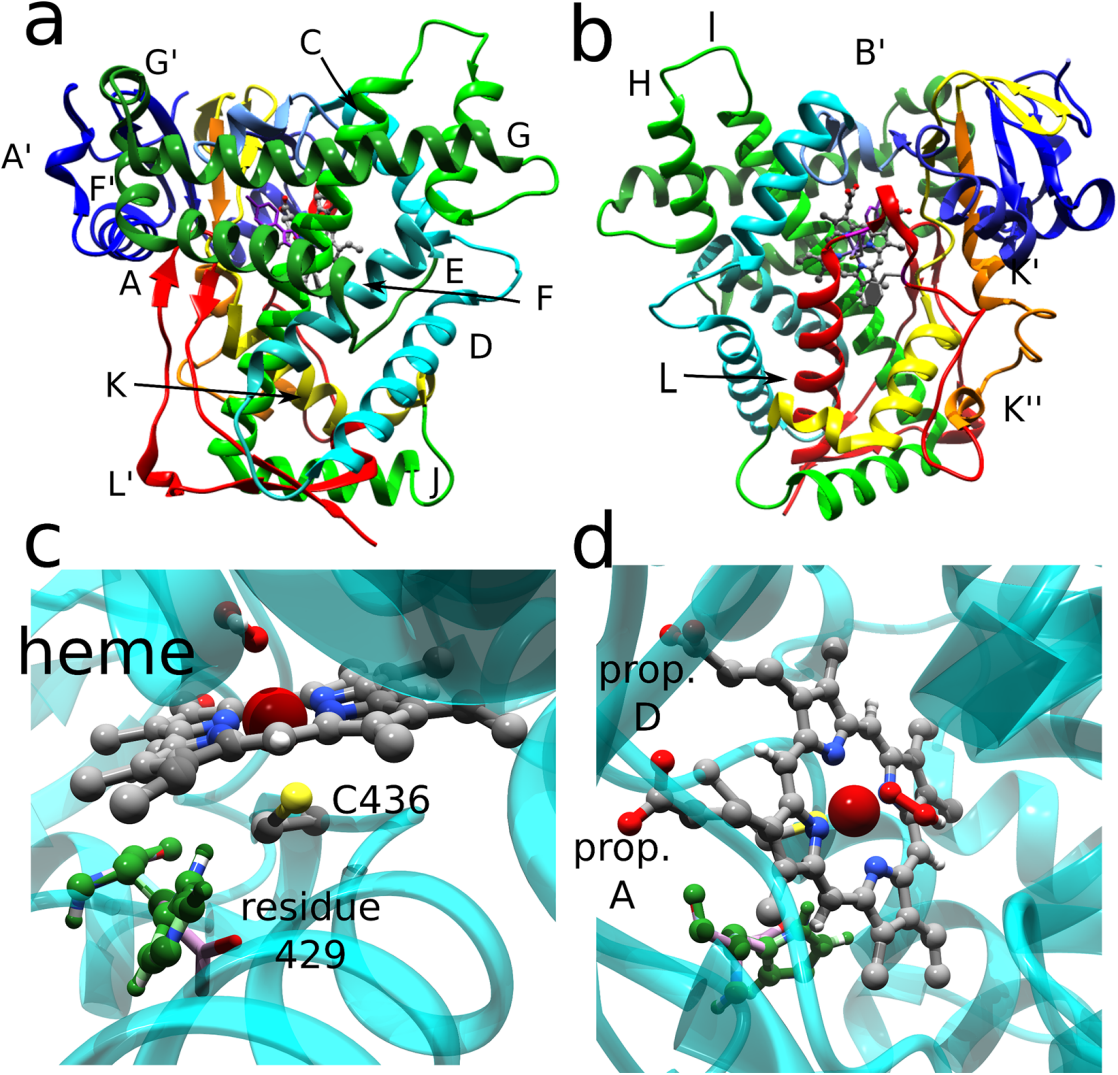
a,b) Cartoon representation of the CYP-2B4 crystal structure (PDB ID 1SUO) with specific domains coloured and labeled according to Scott et al.;[12] the heme group, the peroxide anion and Cys426 are shown as ball and stick. The enzyme is shown looking from the distal (a) or proximal (b) side of the heme group. To be coherent with the literature, the same color map reported in the original experimental work has been used. (c,d) Detail of the active site. The protein backbone is shown as a transparent cyan ribbon, while Cpd0 is shown in and stick style, together with the various rotamers of residue 429. Cys 436, residue 429 and the A and D propionate groups are labeled.

Initial coordinates were obtained by the 1SUO[9] crystal structure (at a resolutions of 1.9 Å, see Fig 2A and 2B). At first, the enzyme was modified in order to form the Cpd 0 species, replacing the inhibitor present in the crystal ctructure with a O-OH covalent moiety.[43] The 4 mutations (Ala, Glu, His and Leu) were generated using the Dunbrack rotamer library[45]. In Fig 2 (panels a, b), we report a pictorial view of the WT form using the original crystal (1SUO.pdb) structure, with various domain shown in different colours; Fig 2C shows the active site with the with the mutated residue 429,which is located at the proximal side of the CYP 2B4 active site, and in particular the region adjacent to the cysteine residue as the fifth ligand to heme iron atom. Missing hydrogen atoms were added by MolProbity[46] and protonation states counter-checked with H^++^ code[47]. Simulations were carried out with GROMACS, version 4.6.6.[48] Afterward, the entire enzyme was placed at the center of a square box of size 10.76 nm, subsequently filled with SPC[49] water molecules at a density of 1000 kg/m^3^; Cl^-^ anions were added to ensure the overall electrical neutrality of each system. The GROMOS 54A7 force field[50] was adopted for modeling the enzymes. Electrostatic interactions were accounted for by means of the Particle Mesh Ewald method[51] using a cutoff of 1.5 nm for the real space and Van der Waals interactions. The LINCS algorithm[52] was used to constrain all bond lengths and angles. Relaxation of solvent molecules and Cl^-^ anions was initially performed keeping solute atoms restrained to their initial positions with a force constant of 1000 kJ/(mol • nm^2^), for 3.0 ns in a NPT ensemble at 1 bar using the Parrinello-Rahman barostat [53] and a coupling constant of 1.0 ps; temperature was increased in 50 K steps from 0 to 298.15 K using the velocity rescale method [54] and a coupling constant of 0.1 ps; the integration time step was increased progressively from 0.1 to 1.0 fs. Each system was carried back to 0 K and then heated again to 298.15 K in a NVT ensemble using the same stepwise fashion (for a total of 3.0 ns of further thermalization) without restrains on protein atoms. The five systems were then simulated for 150 ns each in a NVT ensemble with a time step of 2.0 fs (updating the neighbor list every 10 steps). Additional short (10 ns) simulation were also carried out for each system generating new random velocities. In total, 2 short simulations were carried out for each system. Thus, 3 simulations were carried out for each system or 18 simulations in total. The starting coordinates for these trajectories were selected observing the Root Mean Square Deviation graph (see Section 3.1 and S1 Fig).

### 2.2 Analysis of trajectories

Root mean square deviations (RMSD), root mean square fluctuations (RMSF) and principal components analysis (PCA) were carried out using standard GROMACS analysis tools applying usual definitions[54].

Radial distribution functions of species *B* with respect to species *A* were calculated as:

whereis the particle density of species *B* at distance r around species *A*, and ⟨*ρ*_*B*_⟩_*local*_ is the particle density of type B averaged over all spheres around particle *A* with radius *r*_*max*_.

#### 2.2.1 Artificial trajectories

For cluster analysis and the search of solvent access pathways frames from the various simulations were selected using the following protocol: a trajectory was divided in equal chunks using a previously determined stride (see below); a frame was then selected randomly from each chunk, excluding those within a cutoff of neighbor chunks equal to 10% of the stride. Thus, a stride of 40 ps mean selecting frames randomly in each chunk, excluding those within 4 ps of neighbor chucks. Strides of 40 ps and 80 ps were selected following Shao et al[55]: in this study it was observed, using several different clustering methods, that frames separated by time spans up to 50 ps are usually grouped in the same cluster and thus a lower stride (i.e. a higher number of frames) does not improve the quality of the analysis. This procedure minimized the correlation between frames due collective motions in the 1–10 picoseconds time scale. After this random sampling the frames obtained were concatenated together to form an “artificial trajectory”, always using the following order for systems: WT, F429A, F429E, F429H, F429L. For each system the 2 short simulation were concatenated after the 150 ns one. Note that concatenating the trajectories of the five systems was meant as a way to easily identify and comparing shared conformational basins in the different systems. In this study, whenever frames from multiple simulations have been concatenated and/or analyzed, the resulting trajectory is referred to as an “artificial” trajectory. In particular, 3 frame sets, were created by concatenating the frames from the various simulations. In the first one (hereafter labeled as frame set #1) an average stride of 80 ps was used; the number of frames sampled from each trajectory is given in Section 3.1. This first artificial trajectory was used in order to optimize the number of clusters and/or the cut-off in the pairwise distances in a series of calculation. A second one (hereafter labeled as frame set #2) was created using a stride of 40 ps; this artificial trajectory was used to repeat the clustering, select centroids and perform fluctuation and principal component analysis. A third trajectory (hereafter labeled as frame set #3) was also created, using a stride of 80 and only taking frames from the 150 ns simulations. To avoid the inclusion of configurations from far from equilibrium states in the 150 ns runs, the first part of these simulations was excluded from the generation of artificial trajectories (see Section 3.1). Note that the computational cost for clustering scales as N^2^, where N is the number of configurations used; on the other hand, a larger data set allows to find more representative centroids structures.

#### 2.2.2 Clustering algorithms

Conformations were clustered using Cα and Cβ atoms (thus taking into account side chain orientation) employing a partitioning approach (*k-medoids*) and the so-called GROMOS method[56] and always using pairwise RMSD as a measure of distance between 2 data points (sampled structures). The k-medoids searches for *k* representative objects (the medoids) among the observations in a dataset (the MD frames here). After finding a set of k medoids (randomly select at the beginning), k clusters are constructed by assigning each observation to the nearest medoid (using the pairwise RMSD to measure distances between structures); then the cost of each cluster is calculated swapping every non-medoid point with its medoid and selecting the configuration with the minimum cost (calculated as decrease or increase of variance within each cluster) as a new medoid to build new clusters. The procedure is iterated until there is no change in the medoids. In the GROMOS method,[56] after a distance cut-off is set, the structure with the largest number of neighbors (i.e. configurations within the cutoff range) is taken as the centroid of the first cluster and it is eliminated by the pool with all its neighbors; the process is repeated until all structures have been assigned to a cluster or are singletons; the number of clusters is not fixed.

Independently of clustering, centroids of sub-trajectories were also associated to those frames which maximized the sum of the similarity scores between conformation pairs:

$$C={argmax}_{i}\sum e^{\frac{-{rms}_{ij}}{\sigma }}$$

where $s_{ij}=e^{-{rms}_{ij}}$ is the similarity beween frames *i* and *j*, *rms*_*ij*_ is the RMSD between *i* and *j* and σ is standard deviation of the RMSD matrix.

#### 2.2.3 Clustering internal validation

The k-medoids method naturally aims at minimizing the internal cluster variance thereby maximizing the variance between clusters. When the optimal number of clusters, *n*_*C*_, is unknown a “scan” is often performed changing the *n*_*C*_ parameter and the results obtained are compared by plotting the within- cluster variance (actually the within cluster sum of squares, WSS) as a function of *n*_*C*_. The optimum *n*_*C*_ value is such that adding more clusters does not yield significant variation of WSS; this is the so-called “elbow criterion”. However, sometimes it may be difficult to detect such an “elbow” and the best values of *n*_*C*_. To further assesses the results of the k-medoids procedure we also applied 2 internal evaluation criteria, namely the Dunn index and the Davies-Bouldin (DBI) index [55]. The Dunn index is defined as:

$$DI=\frac{min\delta (C_{i},C_{j})}{max\Delta_{k}}$$

where *Δ*_*k*_ is the distance between centroids in this work and *δ*(*C*_*i*_, *C*_*j*_) is the maximum intra-cluster distance. When testing different pre-determined number of clusters (as in k-medoids) a maximum of *DI* corresponds to the most probable number of clusters. The Davies-Bouldin index is defined as:

$$DBI=\frac{1}{N}\sum \underset{i\neq j}{max}\frac{d_{i}+d_{j}}{d_{ij}}$$

where *N* is the number of clusters, *d*_*i*_ is the distance of elements from the centroid in cluster *i* and *d*_*ij*_ represents the average inter-cluster distance; *DBI* measures the maximal value of within-cluster dispersion as compared to inter-cluster distances and has a minimum value when the number of clusters chosen yields the best combination of compact and well-separated clusters.

#### 2.2.4 Analysis of water pathways

The analysis of the water aqueducts was performed by means of the CAVER 3.1 program[57], as done by Cojocaru et al.[8] Note that we define a “tunnel” is a single solvent path from the protein surface to the selected point existing in a given frame (if any); and a “pathway” as a group of homogeneous tunnels, obtained by a cluster analysis of the tunnels found along the trajectory. Such a cluster *is not* the same as the groups obtained by applying the k-medoids or GROMOS clustering algorithms. Single tunnels are ranked according to their cost, which takes into account the tunnel length and radius along its length and pathways are ranked according to their average throughput (which is simply *e* ^*-cost*^ and ranges from 0 to 1) on tunnels in a given cluster. We used spherical probes of either 0.7 or 1.3 Å and the coordinates of the Sγ atom in C436 or of the center of mass of the peroxide anion as starting points. Hydrogen bond connectivity along the sampling was analyzed as follows: 2 protein residues were considered to be joined by an H-bond in a given MD snapshot if the Donor-Acceptor distance was less than 3.5 Å and the Donor–Hydrogen–Acceptor angle was ≤30°. Unless otherwise specified an automatic cut-off to the lifetime equal to 50% of sampling (75 ns) was applied when discussing the existence of hydrogen bonds.

Data analysis was performed using standard GROMACS tools (RMSD matrices and GROMOS clustering), the Pycluster library (k-medoids) or in house written software. Graphs have been obtained with the Grace program and images have been created using the VMD[58] and Chimera[59] packages.

## Results

### 3.1 Conformational basins of CYP-2B4 and F429 mutations

Mammalian CYP-2B4 is known to adopt both open and close conformations in response to bound substrates and crystallization conditions. We first examined the conformational flexibility of CYP-2B4 in response to single point mutations by monitoring the Root Mean Square Deviation (RMSD) of α-carbon atoms positions with respect to the crystal structure. S1 Fig (left panel) in the Supporting Information (ESI) shows the RMSD calculated from the five 150 ns trajectories as a function of time. It can be observed that all studied systems show a conformational relaxation from the 1SUO structure with increasing RMSD that lasts up to 20/30 ns along the trajectories. Afterward, smaller oscillations of the RMSD were observed, within a range of about 0.2 nm. The spread of RMSD values can also be appreciated looking at histograms in S1 Fig, right panel. Based on the results of RMSD analysis the first 30 ns of all 150 ns trajectories were discarded in the following clustering and water pathways analysis.

Two additional short trajectories of 10 ns were started for each system selecting the starting coordinates at 60 and 130 ns and generating new random velocities at 298.15 K. The RMSD calculated as a function of time from the (10) trajectories is superimposed over the original trajectory in S2 Fig; it may be easily observed that each system continues to sample conformations comparable to those in the originating trajectory, confirming the simulations had reached an acceptable equilibrium. Artificial trajectories were created by selecting frames from 30 to 150 ns as well as all the configurations from the 10 ns simulations for each system (equivalent to 140 ns of sampling for each of the 5 species). Using a 80 ps stride yielded a total of 8750 frames while a 40 ps stride resulted in an artificial trajectory formed by 17500 frames; using only frames from the longer simulations from 30 ns onward and a 80 ps stride yielded an artificial trajectory formed by 7500 frames.

Following the RMSD calculation, the conformational space sampled by the enzyme in all the simulations was explored my means of clustering methods. The first step involved the application of the k medoids method in order to find if and which of the mutated species shared conformational sub-spaces that allowed to group them together, that is to evaluate if there was an optimal number of clusters between 2 and five. Before the actual clustering was performed the distributions of RMSD distances obtained from frame sets #1, #2 and #3 were compared (including the third one, which did not included frames from the 10 ns simulations); the results are shown in S3 Fig; it is easily observable that the distribution of distances (and thus of conformations) obtained from the 3 artificial trajectories is equivalent. To determine the best *n*_*C*_ value the k-medoids algorithm was applied to frame set #1; the analysis of the cluster-wise total variance (see S4 Fig, panel a) indicated 3 clusters as the best solution even if an observable gain is still observed with *n*_*C*_ = 4. Calculation of the DI and DBI evaluation criteria (see Table 1) confirms *n*_*C*_ = 3 as the best choice. S4 Fig (panel b) illustrates the partition of the studied systems in clusters and shows that the F429A, F429E and F429H mutants were assigned to the same cluster while WT, F429L formed distinct classes. Using *n*_*C*_ = 2 yielded one class including bulky hydrophibic side chains (i.e. WT and F429L) and hydrophilic or small ones (F42A, F429H, F429E); with *n*_*C*_ = 3 F429L and WT formed distinct classes and finally *n*_*C*_ = 4 separated F429H from the Ala and Glu mutations.

**Table 1:** K-medoids clustering. For a given *n*_*C*_ (first row) the Dunn index (DI), the Davies-Bouldin index (DBI) and the size of the various clusters are shown. The column showing the best *n*_*C*_ value is typed in italics. Frame set #1 was used in the analysis.

Number of clusters	2	*3*	4	5
**DBI**	0.940	*0*.*839*	0.860	0.841
**DI**	0.910	*1*.*023*	0.834	0.810
**Cluster sizes**				
#1	6295	1750	1750	1750
#2	2451	5243	3429	1738
#3		1753	1749	1761
#4			1818	1749
#5				1748

**Fig 3:**
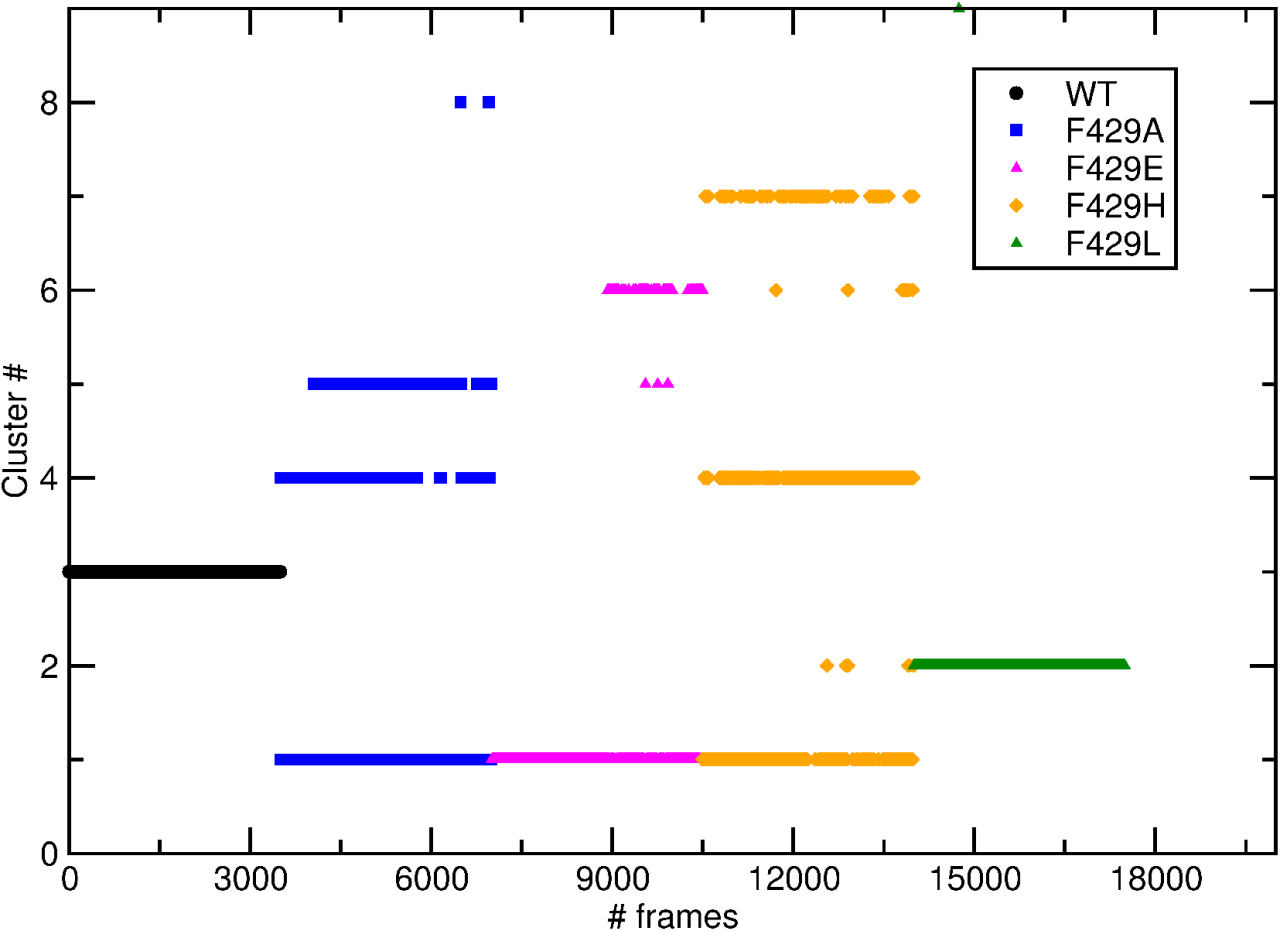
Clustering of CYP2-2B4 conformations. Distribution of structures from 17500 frames artificial trajectory (frame set #2) in clusters (y axis) and simulations (x axis) using GROMOS. Snapshots originally sampled in the 15 trajectories are represented as black circles (WT), blue squares (F429A), magenta triangles (F429E), orange squares (F429H) and green down pointing triangles (F429L).

On the basis of the k-medoids results we ran a scan over the cut-off distance using the GROMOS method to verify if a reasonable choice of this parameter result in 3 statistically representative clusters while filtering out less significant conformations. The RMSD histograms (S2 Fig) feature a first maximum at 0.17 nm and a far higher one at 0.290 nm. Five clustering runs were performed using frame set #1 and cut-off values of 0.18, 0.20, 0.22 0.24 and 0.26 nm, respectively. A cutoff of 0.20 nm created 6 major clusters (13 in total) with no mixing of trajectories. Three major clusters were obtained with 0.22 nm which assigned the systems to classes with the same pattern obtained in k-medoids. Cut-off values of 0.24 and 0.26 nm caused a partial inclusion of WT and F429L configurations in the first (larger by construction) cluster already including the other systems. An additional scanning with an average linkage algorithm and cut-offs values of 0.14 to 0.18 nm yielded the same pattern, i.e. either one, three or five statistically representative clusters with the same correlation between specific simulations and clusters (data not shown). Thus, 0.22 was selected as the best cut-off range and the GROMOS clustering was again applied frame set #2. The results are shown in Fig 3 and S3 Fig in the ESI. Three 3 major clusters were obtained; smaller clusters (from #4 to #6) containing short time transitions were still present for F429A and F429E and F429H.

It is clear that the Ala, Glu and, to a lesser extent, His mutations had a high degree of similarity, the corresponding configurations being located in the biggest cluster, and a relevant conformational freedom since the smaller clusters included mostly configuration from these systems. On the other hand, the Phe, and Leu side chains yielded isolated conformational sub-spaces with little or no overlap between trajectories. This may be further understood by inspecting S5A Fig in the ESI: clusters #1 (6935 frames, the larger by definition; see also S5A Fig) included F429A, F429E and F429H structures; clusters #2 and #3 accounted for 3505 and 3500 structures sampled the WT and F429L simulations. These results may further be observed looking at the transitions between cluster pairs (S5B Fig): the number of transitions between cluster #1 and clusters #5 and #7 is high (see also S3 Fig, panel b) and same is observed between clusters #4 and #6; clusters #2 and #3 on the other hand showed almost no transitions. In summary, all mutants tend to generate trajectories whose conformational spaces differs from that of the wild type enzyme. F429A and F429E displayed an overlap of their conformational spaces while F429H and F429L sampled a distinct one. A comparison between available experimental crystal structures (i.e. 1SUO[43] and 4MGJ[44]) and the centroids of WT (found at 123,2 ns) and F429H (found at 60.68 ns) was made; the two conformation generated by MD simulations had a RMSD distance of 0.0073 nm while their distance from the corresponding experimental structure was 0.0023 (1SUO–WT) and 0.064 (4MGJ–F429H). The distance between centroids is greater than that with the experimental structures showing that the simulation were at least partially able to capture the diversity between the WT and the His mutation; note that the 4MGJ structure has some missing loops. A superimposition of these structures is shown in S6 Fig.

**Fig 4:**
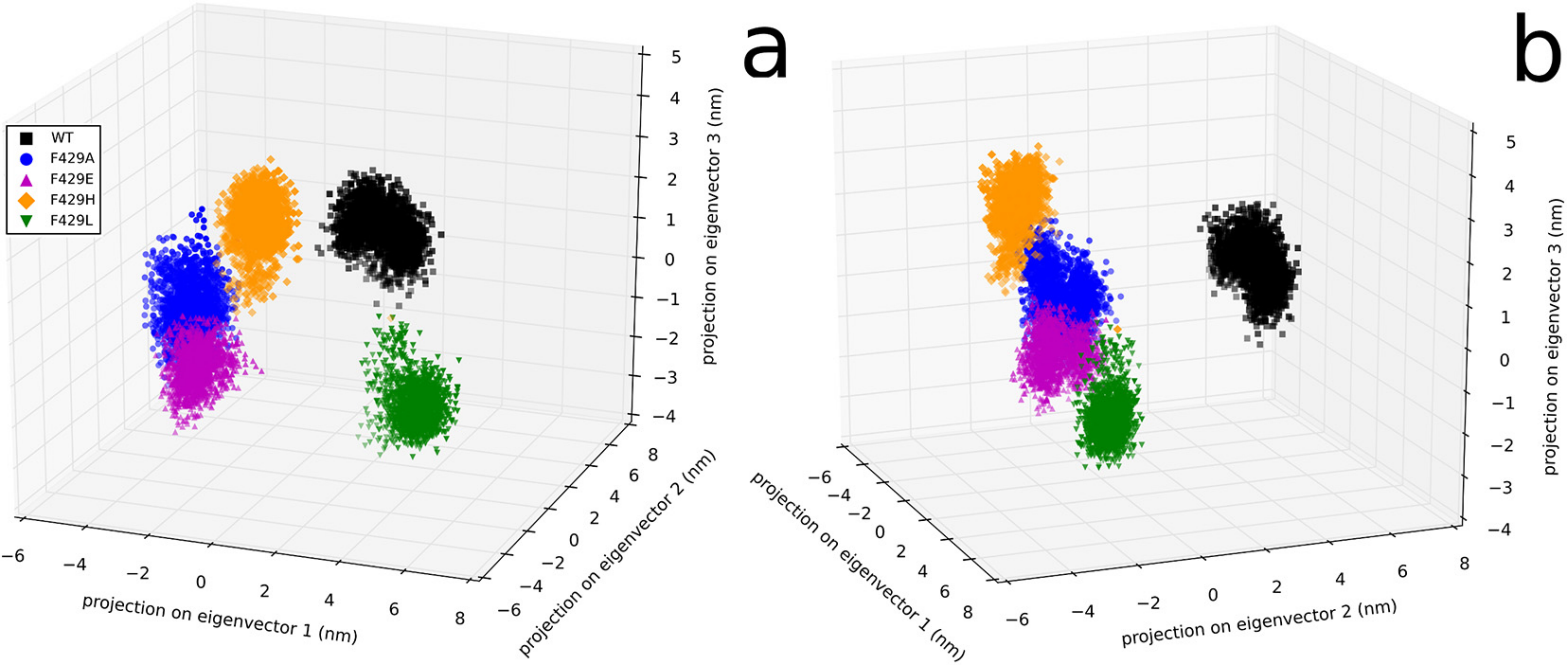
Principal component analysis. Projection of the trajectories of Cα atoms from the larger artificial trajectory in the volume spanned by the first 3 eigenvectors obtained by the principal component analysis, from 2 different points of view. Frames have been represented as dots coloured in black (WT), blue (F429A), magenta (F429E), orange (F429H) and green (F429L).

A principal component analysis of the frame set #2 was performed in order to correlate how much the separation of enzymes in distinct classes could be related to high amplitude motions in the simulations. Fig 4 shows a projection of the clusters over the plane spanned by the eigenvectors (accounting for 75% of total variance). Indeed, conformations pertaining to the systems studied formed conformational basins in this volume which matched almost perfectly the separations obtained by the clustering procedure: WT and F429L were separated by other mutations along the first eigenvector and between themselves along the second one. F429A and F429E were joined a continuous region and were connected (but distinct) to F429H along the third eigenvector.

### 3.2 Structural Fluctuations in clusters

**Fig 5:**
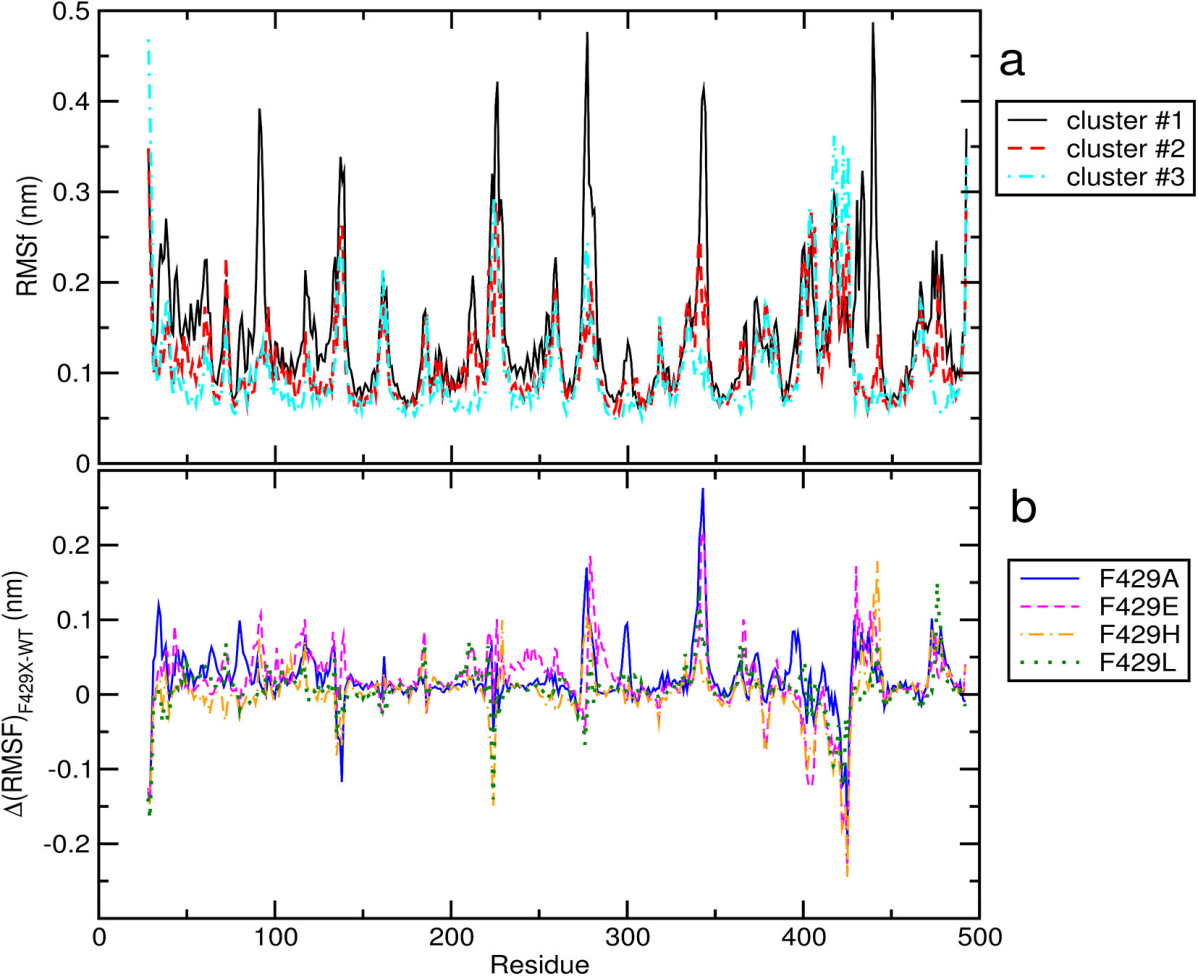
Root Mean Square Fluctuations in clusters and specific systems. **a)** Cα atom RMSF in the 3 clusters obtained by clustering. Cyan dashed line: cluster #1 (i.e. frames from the F429A, F429E and F429H simulations); full black line: cluster #2 (WT); magenta dot-dashed line: cluster #3 (F429L). **b)** difference of RMSF calculated from the trajectories of 4 mutants with respect to the WT trajectory. Full blue line: F429A; magenta dashed line: F429E; orange dot-dashed line: F429H; green dotted line: F429L.

Following the investigation of global structural changes induced by the 4 point mutations with respect to the active site we proceeded with investigating the possible role of single residues in generating these differences, by comparing the Root Mean Square Fluctuation (RMSF) Cα atoms. We have compared in Fig 5 the RMSF calculated obtained from the frames of clusters #1, #2 and #3 (13940 frames in total) trajectories (panel a) with the RMSF *difference* obtained by directly subtracting the RMSF yielded by the wild-type enzyme to those obtained from the 4 mutants trajectories (panel b) (using configurations from frame set #2). Three regions, centered around M137, K225 and G418 showed conserved high fluctuations in all clusters as well as in the original simulations. A number of relevant differences in the fluctuation patterns could be observed; for instance, the first cluster showed larger fluctuations around residues A92, S277, R343 and K433. In general, the region 430–435 near the C436 and the hem distal side features high fluctuations in the Ala/Glu/His mutants as compared to the WT enzyme or F429L. Inspection of panel b shows that in general F429A and F429E showed the greatest variation as compared to WT while F429L produced the most similar fluctuations. It can also be seen that the fluctuation in A92 is mostly due to configurations from the F429E and F429H trajectories while that of S277 and R343 to F429A and F429E; peaks associated to E424 were observable in the WT and F429L trajectories (and thus in clusters #2 and #3).

**Fig 6:**
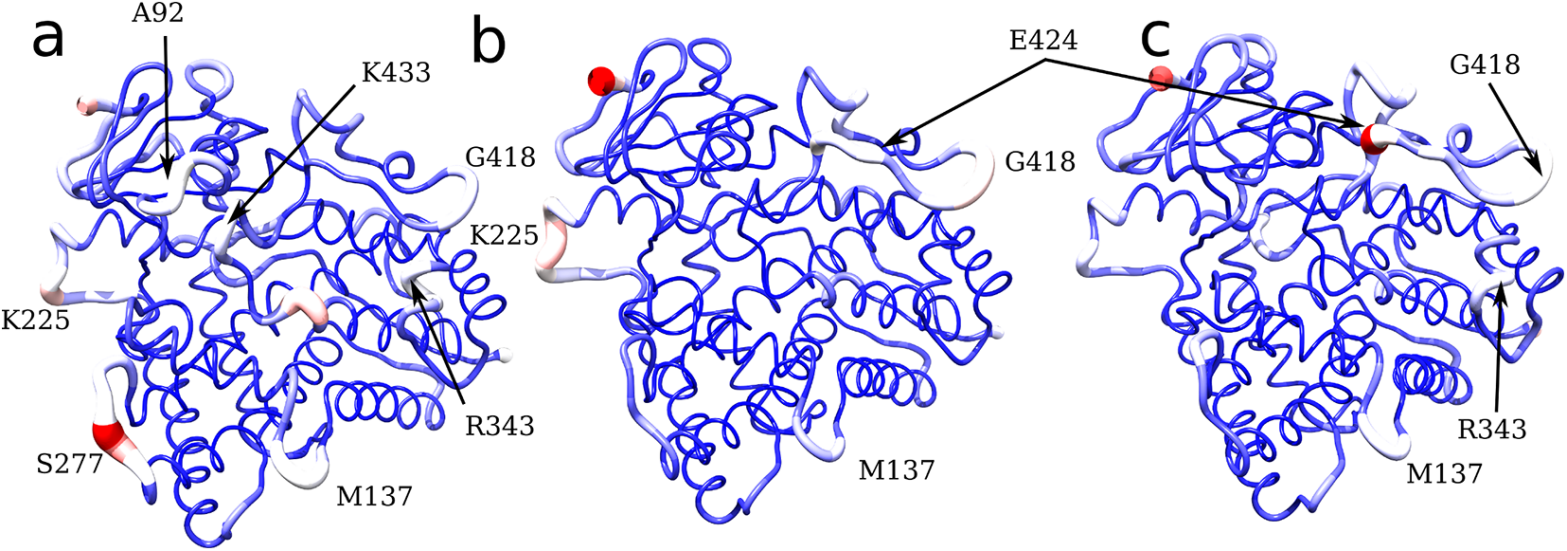
Per residue averaged root mean square fluctuation. Average structures of the 3 clusters, depicted using ribbon colours and thickness proportional to the RMSF. **a)** First cluster (F429A, F429E, F429H). **b)** Second cluster (Wild Type enzyme). **c)** Third cluster (F429L).

Fluctuation patterns observed in the clusters have been mapped on their centroid structures in Fig 6. It is worth to observe that residue S277 is located at the hinge between helices H and I and that K433 is located in loop before helix L.

### 3.3 Water pathways regulation by H bonds

#### 3.3.1 Proxymal side hydration

**Fig 7:**
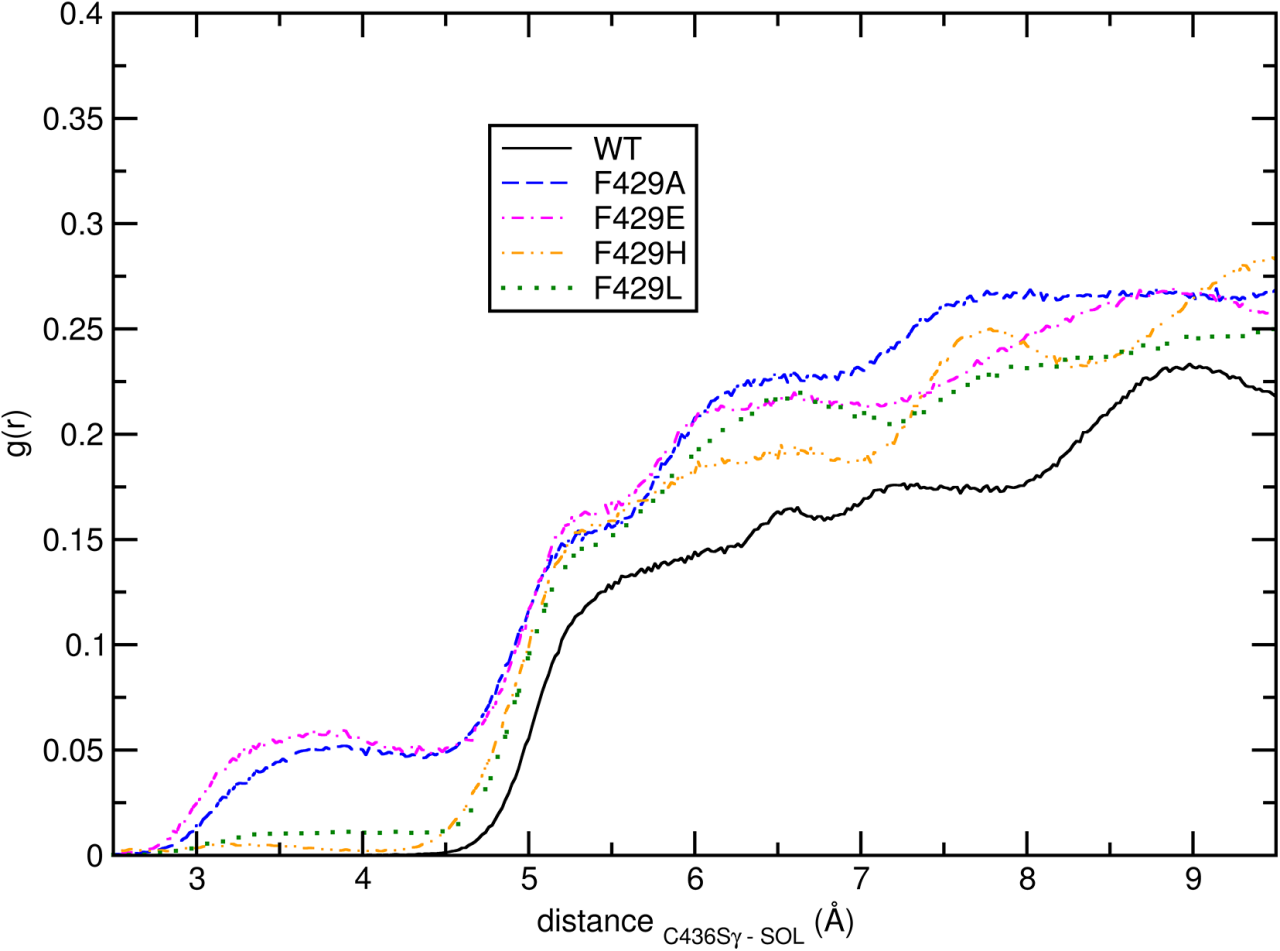
Cys436 hydration. Radial distribution functions of water molecules (center of mass) around the Sγ atom in C436 calculated from the WT (full black line), F429A (blue dashed line), F429E (magenta dot-dashed line), F429H (orange dashed line) and F429L (green dotted line).

Fig 7 shows the radial distribution functions (*g*(*r*)) of water molecules around the Sγ atom of the catalytic residue Cys436. A sharp separation of trajectories in classes is observable: the wild-type enzyme shows the lowest penetration of H_2_O towards the S atom followed by F429H and F429L and then F429A and F429E; using a cut-off of 7 Å this corresponds to a number of water molecules in the neighbourhood of C436 equal to 4 (WT), 7 (F429A and F429E), or 5 (F429H and F429L) water molecules, respectively.

**Fig 8:**
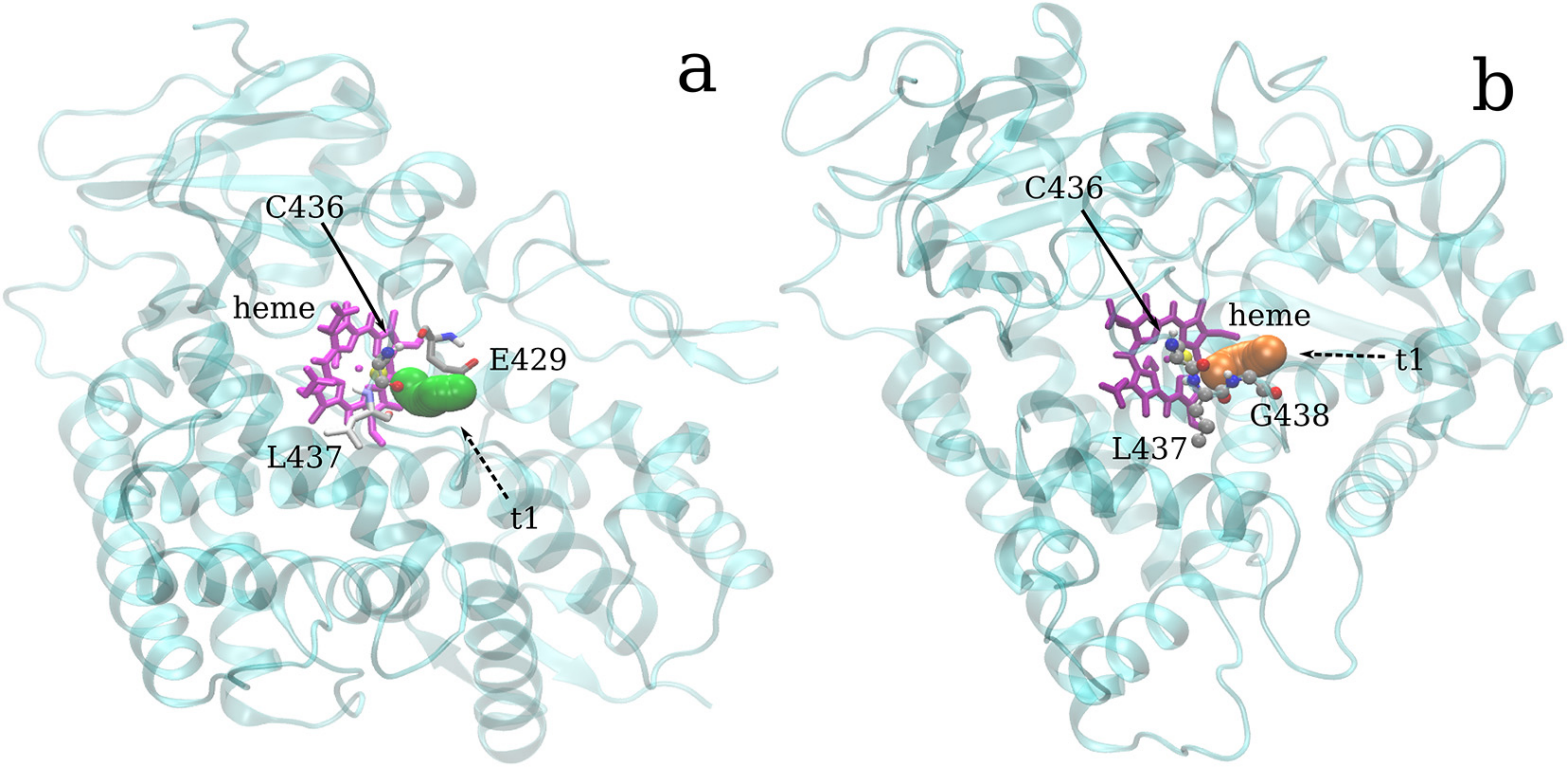
Tunnel towards Cys436. The heme group is shown in purple and the protein backbone as a cyan transparent ribbon. Cys436 is always shown in ball and stick. **a)** Tunnels in the centroid of clusters #1. The bottleneck residues E429 and L437 are represented in licorice. **b)** Tunnel of the F429H simulation; residues G438 and L437 are also shown.

Interestingly, the Leu and His mutants display a similar pattern, apparently at odds with the results of the cluster analysis; however, the pca (Fig 4B) showed that the separation between F429H and F429L share is less as compared to WT, particularly along eigenvectors 2 and 3. This behavior can be explained by looking at representative structures from the 3 clusters. Representative structures were obtained from the three clusters by finding new centroids using the coordinates of the complete protein and the frames forming clusters #1, #2 and #3 (see Section 3.1). The centroid for cluster #1 was found at 36.68 ns in the F429E trajectory; the centroids of clusters #2 and #3 were found at 119.39 and 123.20 ns, in the F429L and WT trajectories, respectively. Inspection of these representative structures by means of the CAVER software showed that in cluster #1 a single partially open pathway (see Table 2) was observed whose bottleneck residues were A/E429, C436 and L437. The orientation of the tunnel with respect to the bottleneck residues in shown in Fig 8A. No open tunnels were found analysing the centroids of clusters #2 and #3, meaning that hydrophobic side chains such as Phe and Leu effectively blocked the access of water molecules to the thiolate. Since the radial distribution functions showed in Fig 7 are not fully coherent with the assignation of the Glu and His mutants to the same class and because the analyzed centroid was sampled from the F429E simulation we decided to analyse also the most representative structure of the F429H alone, which was sampled at 60.68 ns. The results were similar to those obtained for cluster #1 (see Table 2 and S7 Fig in the ESI for a comparison of tunnel radius vs tunnel length) with a slightly decreased throughput and a flat profile and small radius but without real bottlenecks.

**Table 2:** Pathways towards the Sγ atom in C436. The table shows the length, bottleneck radius and throughput. The last column summarizes the simulation(s) in which a given tunnel was observable.

Cluster/system	Bottleneck radius (Å)	Length (Å)	Throughput
Cluster #1	0.86	4.40	0.657
F429H	0.89	5.79	0.570

The tunnel leading to C436 and the thiolate group are lined by residues in the range R434-T444. Looking at differences in the hydrogen bond network (see S1 Table) involving these residues may be useful to explain these results and to relate them to the conformation clustering part. Note that this analysis was carried out on all frames (starting from 30 ns, see section 3.1 and S1 Fig) on the original trajectories, including the short 10 ns simulations, and showed that WT and F429L featured a number of relevant differences in stable hdyrogen bonds as compared to the Ala, Glu and His mutations. For instance, WT lacked the heme-R434 interaction which was very stable in all systems while both WT and F429L lacked the S430-heme interaction. Further, I435-K433 was observed only in featured WT and F429L; L437 featured a different network in WT as compared to all other species. These differences correlate well with the absence of tunnels in both WT and F429L even if they were assigned to different clusters and with the more subtle differences observed between F329A/E and F429H. *Note that the R434—D propionate group and S430—A propionate group hydrogen bonds*, *present with varying stabilities in the mutant species but not in the WT*, *have been also observed in the recent experimental study of F429H*[44].

#### 3.3.2 Distal side hydration

**Fig 9:**
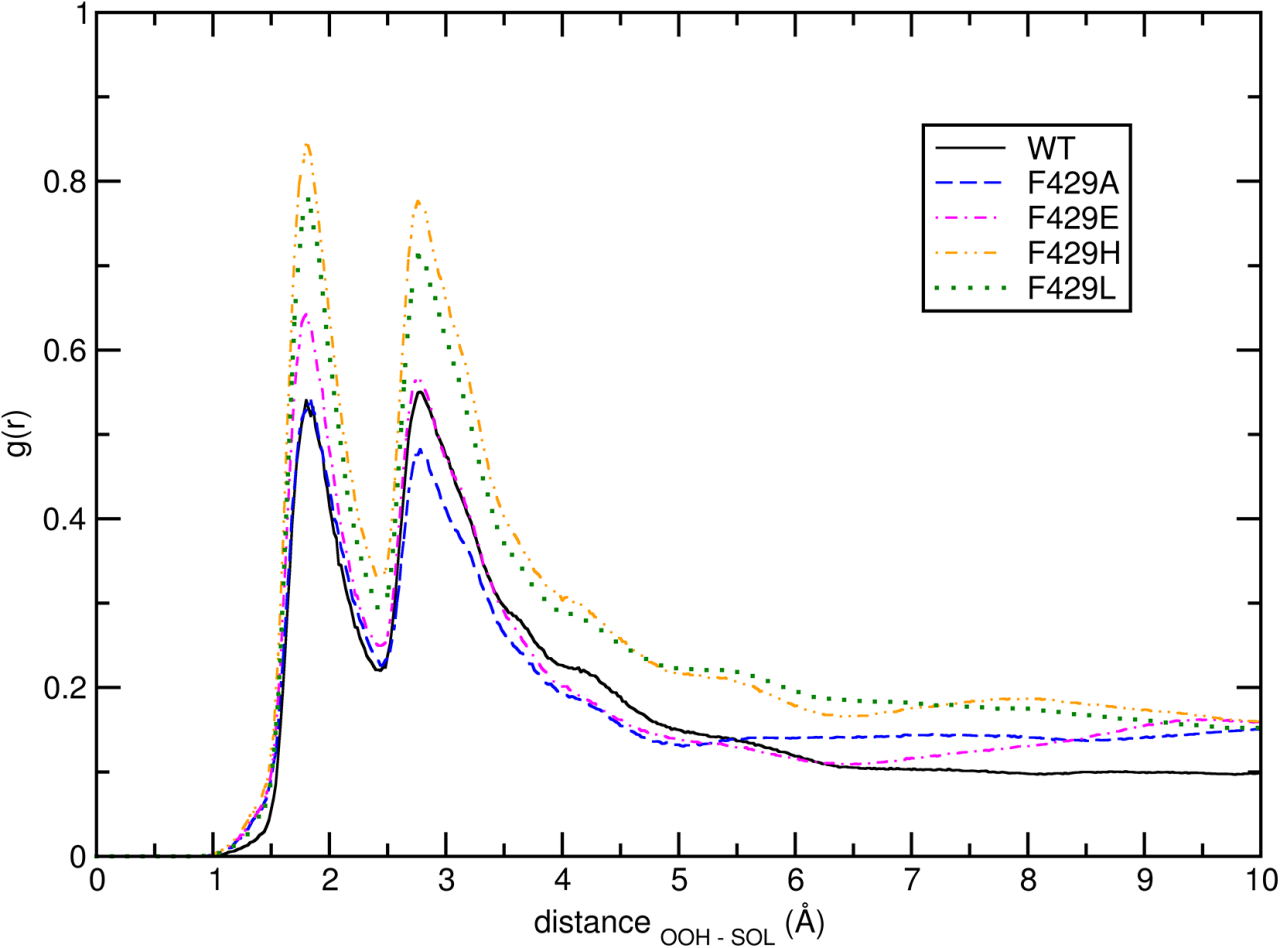
Peroxide anion hydration. Radial distribution function of water molecules (center of mass) around the OOH^-^ anion calculated for the WT, F429A, F429E, F429H and F429L, respectively.

The radial distribution function of water molecules around the peroxide anion is shown in Fig 9:

The *g*(*r*) show the same maxima and minima in positions but different heights and clearly a higher level of hydration as compared to the proximal site: at least 10 water molecules were always present within 7.0 Å. In this case the separation of trajectories in classes is different: relatively comparable radial distribution functions were obtained from the F429H and F429L trajectories and from the F429A and F429E trajectories, with the WT enzyme featuring a *g*(*r*) with intermediate properties as compared to F429A and F429H. To elucidate these results water access pathways have been analysed also for the distal site using CAVER;[57] in this more complex case we extended the analysis to the full artifcial trajectory of 17500 frames (in this case the probe radius was put to 1.3 Å). Each frame was analysed searching for all possible tunnels; then, an average linkage clustering was applied to group single tunnels in pathways. In total four major pathways were found. Table 3 shows the result of this analysis along the 17500 frames (equivalent to 700 ns of concatenated sampling) ns of the artificial trajectory, including the number of frames in which a tunnel cluster was observed and its average length and bottleneck radius (note that the total number of frames in which at least one solvent tunnel was less than the total). Performing the same calculation using 8750 or 15000 frames (i.e. with an average stride of 80 ps or with one or 40 ps using only the longer simulations from 30 ns onward) yielded comparable results (data not shown).

**Table 3:** Pathways properties for frame set #2. The table shows the total number of frames in which a specific tunnel cluster was present, the average length and bottleneck radius and the throughput, i.e. the average of *e* ^*-cost*^ across all frames using the cheapest tunnel in each frame. Pathways are shown from highest to lowest average throughput; average values refers to total number of frames in which a pathways was existent. The last column summarizes the simulation(s) in which a given tunnel cluster was observable; systems for which the pathway was less important are shown in brackets.

Tunnel Cluster	# frames	Average bottleneck radius (Å)	Average length (Å)	Av. throughput	trajectories
1	8001	1.637	19.869	0.645	(WT, F429A), F429E, F429H, F429L
2	2086	1.501	20.784	0.576	(F429A), F429E, F429H, (F429L)
3	1074	1.438	19.388	0.598	(F429A), F429H, F429L
4	1110	1.375	21.458	0.520	WT, F429H

**Fig 10:**
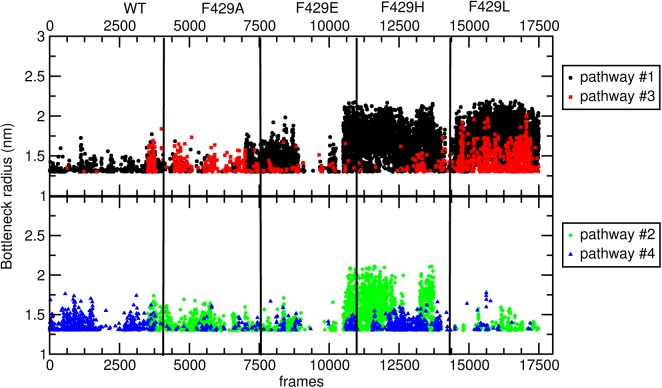
Bottleneck radii of pathways frame set #2. **a)** bottleneck radius of pathways #1 (blue), #5 (orange) and #6 (magenta). **b)** bottleneck radius of pathway #2 (black), #3 (red) and #4 (green). Any given configuration may be easily related to the simulation it belongs to dividing the graph in windows 150 ns wide (see also the graph top).

The partition of simulation in homogeneous groups based on the presence of a specific pathway in a given simulation can be observed in Fig 10, where the bottleneck radii have been plotted as a function of time (using 2 panels to avoid overcrowded graphs).

**Fig 11:**
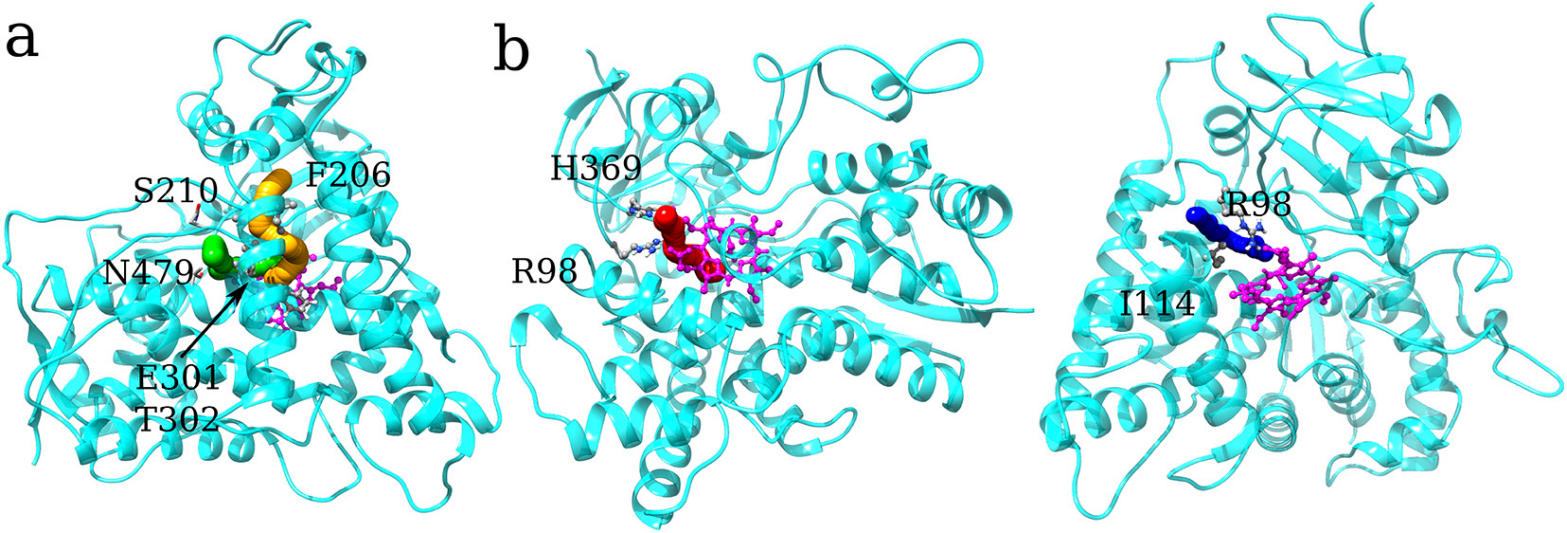
Pathways to the distal site. The heme group, OOH^-^ and Cys436 are shown in magenta. The enzyme and tunnel coordinates shown are those of the corresponding centroid as in Fig 8. **a)** Pathways #1 (red), #2 (green) have been represented; coordinates from the F429H centroid were used. Bottleneck lining residues for pathway #1 and #2 have been represented in ball stick or licorice, respectively. **b)** Pathway #3 (F429L simulation) and corresponding bottleneck residues. **c)** Pathways #4 (WT simulation) and corresponding bottleneck residues.

Table 3 shows that four main pathways for transport of the molecules (including solvent) to the distal site were observed and the frequency and throughput are in good agreement with the *g*(*r*) plot of Fig 9. The first major very wide pathway was sampled, with different frequency, in all trajectories even if the relative frequency and average bottleneck radius observed in the WT configurations was much lower (see Fig 10). In addition, the bottleneck radius obtained in the F429H and F429L simulations (above 1.5 Å) was on average much higher than what observed in other cases. Pathway #2 was mostly a feature of F429H and F429E and to a lesser extent of F429A and F429L. Pathway #3 was observed most often in F429H and F429L. Pathway #4 was sampled mostly in the WT simulation and less frequently in F429H. The overall picture is similar to what observed about the hydration of the proximal site: the three trajectories forming cluster #1 show a varying number of common features between them. They also show feature the pathway with highest average throughput. There is also a degree of similarity between F429L and F429H which agrees with the results of clustering and PCA (see Figs 3 and 4). It is worth observing the distinct behavior of WT which shares just pathway #4 with another system. To understand the origin of access to different water pathways, an atomistic view of the relative positions of these residues is given in Fig 11 where the tunnels and the corresponding bottleneck lining residues are shown.

Access of molecules to the active site of CYP 2B4 through pathway #1 and #2 is regulated by a gate formed by E301 and T302; however pathway #1 is also regulated by S210 (side chain), F206 (backbone) and I209 while pathway #2 by 305 and N479. Note that F202, F206 and S210 formed a networks of hydrogen bonds (see S1 Table) which was very stable in all mutant species but only episodically observed in the wild-type simulation. Very stable bonds between involving the critical residues E301 (with 308) and T302 (the latter with the peroxide anion) were also unique features of WT and it worth to highlight the critical role of these residues in the CYP-2B4 catalytic cycle, as explained by Usharani et al. [43]. The bottleneck of pathway #3 was formed by R98 and I114. Access via pathway #4 was regulated by R98, H369 and R434; again, it is worth to observe the different behavior showed by this residues in the WT simulation as compared to the mutants in general, even if this pathway was relevant also in the F429H trajectory. Note that, like pathways #1 and #2, pathways #3 and #4 featured some common bottleneck residues which had different hydrogen bonds character (see S1 Table) and were observed in different systems (e.g. #3 was sampled in the F429L trajectories and #4 in WT trajectories, respectively).

## Discussion

The key features of the F429H mutant elucidated in the previous computational works[43] were: (a) the role of the Phe alanine in protecting the sulphur atom in C436 and the electrostatic environment around the thiolate group and (b) the interaction between T302 and the peroxide anion which favoured the attack on the meso position. However, the reaction model proposed for the heme-oxygenase behaviour of F429H was primarily based on the wise application of MD results, particularly of PCA,[60,61], in selecting candidate structures for higher level of calculations (e.g. QM/MM). Thus, the obvious direction for further investigation was to extend the simulation time and study additional mutations. To this end, in this study we performed new simulations of both the WT and F429H species extending the simulation time to 150 ns and added equally long simulations of three mutations: the bulky and hydrophobic Phe side chain was changed with Ala (small side chain), Glu (hydrophilic side chain) and Leu (bulky and hydrophobic side chain). This wide range of amino acid properties was studied to address the specific properties of either the WT enzyme or its F429H mutant with respect to other species and thus provide further insights in the function of the Phe residue (a highly conserved one) and of the specific alterations induced by the presence of the imidazole moiety in the mutation.

### Mutation of F429 deeply affects global mechanical properties

As a first step (sections 3.1 and 3.2 and section S3.1 in the ESI) we investigated the amount by which, upon mutation of F429 into another amino-acid, the collective motions of the CYP-2B4 enzyme were affected. Indeed, the results show that the presence of the Phe residue is critical for modulating the structural flexibility of the enzyme since the WT trajectory was always separated from the other systems in clustering procedures and had the highest propensity to form a compact cluster when a cut-off (GROMOS and linkage methods) was used, i.e. no configurations of the WT enzyme were present in the smaller clusters. The results obtained in this first part provided both a tool of simplification (in analysing structural fluctuations) and a key of interpretation (in studying water pathways) of the subsequent results. This results was also confirmed by the principal component analysis of the artificial trajectory used in the clustering. In addition, we observed a different fluctuation profile of residues in the region 430–435 which are critical in the regulation of water pathways to both the distal and proximal sides. The results obtained in this first part can be summarized in the following way: mutation of F429 creates a perturbation in the inter domain weak interactions regulating enzyme fluctuations; this perturbation does create a sharp separation between the WT and mutant species which can be roughly ordered in function of the mutated residue hydrophobicity from Leu to His to Glu and Ala (where Ala is not hydrophobic by itself but does not create enough steric hindrance).

### The Cys436 residue is exposed to solvent in all mutant species

In section 3.3, we analysed the access pathways to both sides of heme group. As remarked in the introduction, controlled hydration/dehydration of the active site is critical for starting the enzyme critical activity and water clusters also play a fundamental role in the heme-oxygenase activity of F429H; in addition, direct interaction with water may provide the same function of the imidazole hydrogen in altering the enzyme function. Thus, any relevant alteration in the hydration properties of the distal and proximal sides of the heme group are likely to have a profound effect on the enzyme activity. The analysis of the proximal side yielded a clear and simple message: mutation of the Phe residue with a less hydrophobic species may open a short and direct tunnel from the protein surface to the proximal side. Such an opening was reduced for the His mutation and greater for Ala and Glu, a separation that is in very good agreement with the classification of systems performed in Section 3.1.

### The F206-E301 pathway is highly affected in all mutant species

The analysis of tunnels for the distal site showed the unique properties of the WT enzyme that displayed a distinct pattern of hydrogen bonds and correlated water pathways. It has been previously proposed[8,11] that this pathway and the CYP’s conformational plasticity that closes and opens it is a key factor to explain the ability of this enzyme to host different substrates and create a proper reaction environment. In a communication by Shah et al.[62], it was shown that in the F297A mutant of CYP-2B4 a reorientation of phenylalanine residues along this pathway, in particular F202 and F203 was observed and this reorientation was at the origin of different poses obtained by docking drugs such as clopidogrel in the WT and mutant enzyme. It is important to observe than the F202 and F206 residues followed completely different dynamics in the WT simulation as compared to the mutant species. It is also worth to remark that in CYP-3A4, the F206 residue is replaced by R212 i.e. a completely different amino acid; nevertheless, the transport of solvent and drugs to active site is still regulated by this residue.[63]

### The solvent pathway via H369 is either closed (F29A and F429E) or altered (F429L and F429H) in mutants

We want to highlight one last feature of the WT system i.e. the presence of a reversible pathway (#4) regulated (among others) by residue H369, absent in the Ala, Glu or His mutants. This aqueduct and its functioning have been studied previously[16], also in other cytochrome species and in the presence of reductase with CYP-3A4 by means of classical molecular dynamics simulations.[13] It has been found that in CYP-3A4 these residues can create a reversible gating for water, together with R375 in the 1TQM structure,[64] which is replaced by H369 in CYP 2B4. Residues H369, K433 and R434 (i.e. R440 in the 1TQM structure) are bottleneck lining residues in pathway #4 (mainly sampled in the WT trajectory). Please note that, K433 showed a rather different mechanical behaviour in conformational clusters (see Fig 5). The different dynamics of these residues in F429H and (more frequently) F429L gave raise to pathway #3.

## Conclusions

Fifteen MD simulations, were carried out to study the conformational behavior of aqueous CYP 2B4 and the F429A, F429E, F429H and F429L single polymorphisms while hosting ferric hydroperoxide (Cpd 0) key intermediate. Three trajectories were generated for each system and sampled for either for 150 or for 10 ns, starting from equilibrated structures and assiging new velocities. In total, each system was simulated for 170 ns; analysis of RMSD prompted us to discard the firsy 30 ns of the longer trajectories yielding 140 of sampling available to data analysis for each system. Comparison of the classical trajectories shed some light on the subtle role played by the highly conserved Phe429 proximal residue in the CYP 2B4 biological machinery. The combined use of different clustering techniques, analysis of collective motion hydrogen bonds and solvent aqueducts allowed us to isolate the most relevant conformational reservoirs of the five systems, highlighting their shared and distinct features. The results showed that, the effect of the Phe429 mutations are propagated across the entire CYP 2B4 structure, altering both protein dynamics and functional access channels to the active site. These alterations follow a pattern that may be related to the mutated residue hydrophobicity (or lack thereof) as compared to the phenylalanine side chain. We conclude, then, that the proximity of Phe429 to the proximal side of CYP-2B4 has a twofold critical function: (*i*) it creates a closed environment for the thiolate group, protecting it from possible oxidation, protonation or other process by neighbour residues or solvent molecules (*ii*) it stabilizes the hydrogen bond network around the active site and, as a consequence, the proper functioning of the access and egress pathways to the heme distal site which are critical for allocating substrate and solvent molecules and thus for the enzyme catalytic activity. We are confident that the presence results might provide a useful starting point for future studies aimed to clarify, how the enzyme plasticity, one of its key features, is affected by single polymorphisms.

## Supporting Information

S1 Fig Root Mean Square Deviation. Left panel: root mean square deviation of Cα carbon atoms yielded by the five trajectories (WT, F429A, F429E, F429H, F429L) as a function of time. Right panel: corresponding probability density histograms (each histogram is built from 1000 equally spaced bins) for each simulation.
(TIFF)

S2 Fig Root Mean Square Deviation of short (10 ns) simulations. The RMSD of each new simulation starting either at 60 or 130 ns is superimposed on that of the originating simulation and drawn using a darker hue. Upper panel: results for the WT and F429E simulations. Lower panel: results for the F429A, F429H and F429L simulations.
(TIFF)

S3 Fig RMSD histograms. Pair wise RMS distances obtained by random selections of frames from the five trajectories using an average time interval of 80 ps (black full line), i.e. frame set #1, 40 ps (red dashed line) using all sets of 3 simulations for each system (frame set #2) and 40 ps using all configurations from 30 to 150 ns in the longer trajectories (frame set #3, green dot-dashed line).
(TIFF)

S4 Fig K-medoids clusters. **a) W**ithin-cluster sum of squares shown as a function of the number of clusters, used for the “elbow criterion”. **b)** Distribution of structures from frame set #1 in clusters (y axis) and simulations (x axis) using k medoids and four clusters. Snapshots originally sampled in the five systems are represented as black circles (WT), blue squares (F429A), magenta triangles (F429E), orange diamonds (F429H) and green down pointing triangles (F429L).
(TIFF)

S5 Fig Cluster sizes and transitions. **a)**: cluster sizes from the analysis of frame set #2 (17500 snapshots, average stride of 40 ps) using a cut-off of 0.21 nm; the first 6 clusters are shown. **b)**: number of transition to or from each cluster; the first 6 clusters are shown.
(TIFF)

S6 Fig Superimposition of the centroids and 4MGJ crystal structures. The centroid of WT (blue ribbons), and F429H (cyan ribbons) simulations together with the 1SUO (orange ribbons) and 4MGJ (light green ribbons).
(TIFF)

S7 Fig Cys436 solvent tunnels. Water tunnel radius as a function of length from the position of Sg in Cys 436. Results are shown from the centroid of clusters #1 (centroid sampled at xx ns in the F429E simulation) and for F429H (centroid sampled at 60.68 ns)**.**
(TIFF)

S1 Table Differences in Hydrogen bond networks in the WT, F429A, F429E, F429H and F429L trajectories. The system in which a specific bond was observed, the donor and acceptor atom and the relative stability (with respect to a total of 140 ns) are shown. “heme-A” or “heme-D” stand for heme-priopionate group A or D (see Fig 2). Interactions are shown if the lifetime is grater than 60% of used configurations (i.e. 91 ns of sampling) Atom names N/O without further specification indicate backbone atoms; N* and O* for Arg and Glu or Asp residues indicate either nitrogen or oxygen atom of the side chain; the sum of the interactions is shown.
(DOCX)

## References

[1] Guengerich FP. Common and Uncommon Cytochrome P450 Reactions Related to Metabolism and Chemical Toxicity. Chem Res Toxicol. 2001;14: 611–650. 10.1021/tx0002583 11409933

[2] Ortiz de Montellano PR, editor. Cytochrome P450: structure, mechanism, and biochemistry 3rd ed. New York: Kluwer Academic/Plenum Publishers; 2005.

[3] Bertz RJ, Granneman GR. Use of In Vitro and In Vivo Data to Estimate the Likelihood of Metabolic Pharmacokinetic Interactions: Clin Pharmacokinet. 1997;32: 210–258. 10.2165/00003088-199732030-00004 9084960

[4] Sono M, Roach MP, Coulter ED, Dawson JH. Heme-Containing Oxygenases. Chem Rev. 1996;96: 2841–2888. 10.1021/cr9500500 11848843

[5] Coon MJ. CYTOCHROME P450: Nature’s Most Versatile Biological Catalyst. Annu Rev Pharmacol Toxicol. 2005;45: 1–25. 10.1146/annurev.pharmtox.45.120403.100030 15832443

[6] Denisov IG, Makris TM, Sligar SG, Schlichting I. Structure and Chemistry of Cytochrome P450. Chem Rev. 2005;105: 2253–2278. 10.1021/cr0307143 15941214

[7] Dawson JH, Sono M. Cytochrome P-450 and chloroperoxidase: thiolate-ligated heme enzymes. Spectroscopic determination of their active-site structures and mechanistic implications of thiolate ligation. Chem Rev. 1987;87: 1255–1276. Available: http://pubs.acs.org/doi/abs/10.1021/cr00081a015

[8] Cojocaru V, Winn PJ, Wade RC. The ins and outs of cytochrome P450s. Biochim Biophys Acta BBA—Gen Subj. 2007;1770: 390–401. 10.1016/j.bbagen.2006.07.005 16920266

[9] Scott EE, White MA, He YA, Johnson EF, Stout CD, Halpert JR. Structure of Mammalian Cytochrome P450 2B4 Complexed with 4-(4-Chlorophenyl)imidazole at 1.9-A Resolution: INSIGHT INTO THE RANGE OF P450 CONFORMATIONS AND THE COORDINATION OF REDOX PARTNER BINDING. J Biol Chem. 2004;279: 27294–27301. 10.1074/jbc.M403349200 15100217

[10] Poulos TL, Finzel BC, Howard AJ. High-resolution crystal structure of cytochrome P450cam. J Mol Biol. 1987;195: 687–700. 3656428 10.1016/0022-2836(87)90190-2

[11] Poulos TL. Cytochrome P450 flexibility. Proc Natl Acad Sci. 2003;100: 13121–13122. 14597705 10.1073/pnas.2336095100 PMC263725

[12] Scott EE, He YA, Wester MR, White MA, Chin CC, Halpert JR, et al An open conformation of mammalian cytochrome P450 2B4 at 1.6-\AA resolution. Proc Natl Acad Sci. 2003;100: 13196–13201. 14563924 10.1073/pnas.2133986100 PMC263748

[13] Fishelovitch D, Shaik S, Wolfson HJ, Nussinov R. How Does the Reductase Help To Regulate the Catalytic Cycle of Cytochrome P450 3A4 Using the Conserved Water Channel? J Phys Chem B. 2010;114: 5964–5970. 10.1021/jp101894k 20387782 PMC2861407

[14] Shaik S, Kumar D, de Visser SP, Altun A, Thiel W. Theoretical Perspective on the Structure and Mechanism of Cytochrome P450 Enzymes ^†^ . Chem Rev. 2005;105: 2279–2328. 10.1021/cr030722j 15941215

[15] Davydov R, Makris TM, Kofman V, Werst DE, Sligar SG, Hoffman BM. Hydroxylation of Camphor by Reduced Oxy-Cytochrome P450cam: Mechanistic Implications of EPR and ENDOR Studies of Catalytic Intermediates in Native and Mutant Enzymes. J Am Chem Soc. 2001;123: 1403–1415. 10.1021/ja003583l 11456714

[16] Oprea TI, Hummer G, García AE. Identification of a functional water channel in cytochrome P450 enzymes. Proc Natl Acad Sci. 1997;94: 2133–2138. Available: http://www.pnas.org/content/94/6/2133.short 9122160 10.1073/pnas.94.6.2133 PMC20053

[17] Loida PJ, Sligar SG. Molecular recognition in cytochrome P-450: Mechanism for the control of uncoupling reactions. Biochemistry (Mosc). 1993;32: 11530–11538. 10.1021/bi00094a009 8218220

[18] Sligar SG, Gunsalus IC. Proton coupling in the cytochrome P-450 spin and redox equilibriums. Biochemistry (Mosc). 1979;18: 2290–2295. 10.1021/bi00578a024 444456

[19] Raag R, Poulos TL. The structural basis for substrate-induced changes in redox potential and spin equilibrium in cytochrome P-450CAM. Biochemistry (Mosc). 1989;28: 917–922. 10.1021/bi00428a077 2713354

[20] Harris D, Loew G. Determinants of the spin state of the resting state of cytochrome P450cam. J Am Chem Soc. 1993;115: 8775–8779. Available: http://pubs.acs.org/doi/abs/10.1021/ja00072a034

[21] Lonsdale R, Oláh J, Mulholland AJ, Harvey JN. Does Compound I Vary Significantly between Isoforms of Cytochrome P450? J Am Chem Soc. 2011;133: 15464–15474. 10.1021/ja203157u 21863858 PMC3180200

[22] Newcomb M, Zhang R, Chandrasena REP, Halgrimson JA, Horner JH, Makris TM, et al Cytochrome P450 Compound I. J Am Chem Soc. 2006;128: 4580–4581. 10.1021/ja060048y 16594688 PMC2536593

[23] Kumar D, Hirao H, de Visser SP, Zheng J, Wang D, Thiel W, et al New features in the catalytic cycle of cytochrome P450 during the formation of compound I from compound 0. J Phys Chem B. 2005;109: 19946–19951. 10.1021/jp054754h 16853579

[24] Isobe H, Yamanaka S, Okumura M, Yamaguchi K, Shimada J. Unique Structural and Electronic Features of Perferryl–Oxo Oxidant in Cytochrome P450. J Phys Chem B. 2011;115: 10730–10738. 10.1021/jp206004y 21812482

[25] Bathelt CM, Zurek J, Mulholland AJ, Harvey JN. Electronic structure of compound I in human isoforms of cytochrome P450 from QM/MM modeling. J Am Chem Soc. 2005;127: 12900–12908. 10.1021/ja0520924 16159284

[26] Zheng J, Wang D, Thiel W, Shaik S. QM/MM Study of Mechanisms for Compound I Formation in the Catalytic Cycle of Cytochrome P450cam. J Am Chem Soc. 2006;128: 13204–13215. 10.1021/ja063439l 17017800

[27] Meunier B, de Visser SP, Shaik S. Mechanism of oxidation reactions catalyzed by cytochrome p450 enzymes. Chem Rev. 2004;104: 3947–3980. 10.1021/cr020443g 15352783

[28] Lonsdale R, Harvey JN, Mulholland AJ. Compound I Reactivity Defines Alkene Oxidation Selectivity in Cytochrome P450cam. J Phys Chem B. 2010;114: 1156–1162. 10.1021/jp910127j 20014756

[29] Sheng X, Zhang H, Im S-C, Horner JH, Waskell L, Hollenberg PF, et al Kinetics of Oxidation of Benzphetamine by Compounds I of Cytochrome P450 2B4 and Its Mutants. J Am Chem Soc. 2009;131: 2971–2976. 10.1021/ja808982g 19209859 PMC2765530

[30] Shaik S, Milko P, Schyman P, Usharani D, Chen H. Trends in Aromatic Oxidation Reactions Catalyzed by Cytochrome P450 Enzymes: A Valence Bond Modeling. J Chem Theory Comput. 2011;7: 327–339. 10.1021/ct100554g 26596155

[31] Cohen S, Kozuch S, Hazan C, Shaik S. Does Substrate Oxidation Determine the Regioselectivity of Cyclohexene and Propene Oxidation by Cytochrome P450? J Am Chem Soc. 2006;128: 11028–11029. 10.1021/ja063269c 16925412

[32] Kumar D, Sastry GN, de Visser SP. Axial Ligand Effect On The Rate Constant of Aromatic Hydroxylation By Iron(IV)–Oxo Complexes Mimicking Cytochrome P450 Enzymes. J Phys Chem B. 2012;116: 718–730. 10.1021/jp2113522 22132821

[33] Eldik R van, Ivanović-Burmazović I. Inorganic/bioinorganic reaction mechanisms [Internet]. Amsterdam; Boston: Academic Press; 2012. Available: http://www.123library.org/book_details/?id=46714

[34] Lian P, Li J, Wang D-Q, Wei D-Q. Car–Parrinello Molecular Dynamics/Molecular Mechanics (CPMD/MM) Simulation Study of Coupling and Uncoupling Mechanisms of Cytochrome P450cam. J Phys Chem B. 2013;117: 7849–7856. 10.1021/jp312107r 23742631

[35] Altarsha M, Benighaus T, Kumar D, Thiel W. How is the Reactivity of Cytochrome P450cam Affected by Thr252X Mutation? A QM/MM Study for X = Serine, Valine, Alanine, Glycine. J Am Chem Soc. 2009;131: 4755–4763. 10.1021/ja808744k 19281168

[36] Altarsha M, Benighaus T, Kumar D, Thiel W. Coupling and uncoupling mechanisms in the methoxythreonine mutant of cytochrome P450cam: a quantum mechanical/molecular mechanical study. JBIC J Biol Inorg Chem. 2010;15: 361–372. 10.1007/s00775-009-0608-3 20225401 PMC2830628

[37] Gunsalus IC, Pederson TC, Sligar SG. Oxygenase-Catalyzed Biological Hydroxylations. Annu Rev Biochem. 1975;44: 377–407. 10.1146/annurev.bi.44.070175.002113 806252

[38] Danielson PB. The cytochrome P450 superfamily: biochemistry, evolution and drug metabolism in humans. Curr Drug Metab. 2002;3: 561–597. Available: http://www.ingentaconnect.com/content/ben/cdm/2002/00000003/00000006/art00001 12369887 10.2174/1389200023337054

[39] Wrighton SA, Schuetz EG, Thummel KE, Shen DD, Korzekwa KR, Watkins PB. THE HUMAN CYP3A SUBFAMILY: PRACTICAL CONSIDERATIONS1*. Drug Metab Rev. 2000;32: 339–361. 10.1081/DMR-100102338 11139133

[40] Guengerich FP, Shimada T. Oxidation of toxic and carcinogenic chemicals by human cytochrome P-450 enzymes. Chem Res Toxicol. 1991;4: 391–407. 10.1021/tx00022a001 1912325

[41] Shimada T, Kim D, Murayama N, Tanaka K, Takenaka S, Nagy LD, et al Binding of Diverse Environmental Chemicals with Human Cytochromes P450 2A13, 2A6, and 1B1 and Enzyme Inhibition. Chem Res Toxicol. 2013;26: 517–528. 10.1021/tx300492j 23432429 PMC4026275

[42] Davydov R, Razeghifard R, Im S-C, Waskell L, Hoffman BM. Characterization of the Microsomal Cytochrome P450 2B4 O _2_ Activation Intermediates by Cryoreduction and Electron Paramagnetic Resonance ^†^ . Biochemistry (Mosc). 2008;47: 9661–9666. 10.1021/bi800926x PMC2709102 18700729

[43] Usharani D, Zazza C, Lai W, Chourasia M, Waskell L, Shaik S. A Single-Site Mutation (F429H) Converts the Enzyme CYP 2B4 into a Heme Oxygenase: A QM/MM Study. J Am Chem Soc. 2012;134: 4053–4056. 10.1021/ja211905e 22356576 PMC3326604

[44] Yang Y, Zhang H, Usharani D, Bu W, Im S, Tarasev M, et al Structural and Functional Characterization of a Cytochrome P450 2B4 F429H Mutant with an Axial Thiolate–Histidine Hydrogen Bond. Biochemistry (Mosc). 2014;53: 5080–5091. 10.1021/bi5003794 PMC4131899 25029089

[45] Dunbrack RL Jr. Rotamer Libraries in the 21st Century. Curr Opin Struct Biol. 2002;12: 431–440. 10.1016/S0959-440X(02)00344-5 12163064

[46] Chen VB, Arendall WB, Headd JJ, Keedy DA, Immormino RM, Kapral GJ, et al *MolProbity*: all-atom structure validation for macromolecular crystallography. Acta Crystallogr D Biol Crystallogr. 2010;66: 12–21. 10.1107/S0907444909042073 20057044 PMC2803126

[47] Gordon JC, Myers JB, Folta T, Shoja V, Heath LS, Onufriev A. H++: a server for estimating pKas and adding missing hydrogens to macromolecules. Nucleic Acids Res. 2005;33: W368–W371. 10.1093/nar/gki464 15980491 PMC1160225

[48] Pronk S, Pall S, Schulz R, Larsson P, Bjelkmar P, Apostolov R, et al GROMACS 4.5: a high-throughput and highly parallel open source molecular simulation toolkit. Bioinformatics. 2013;29: 845–854. 10.1093/bioinformatics/btt055 23407358 PMC3605599

[49] Berendsen HJC, Postma JPM, Van Gunsteren WF, Hermans J. Interaction models for water in relation to protein hydration. Intermolecular Forces. 1981;11: 331–342.

[50] Schmid N, Eichenberger AP, Choutko A, Riniker S, Winger M, Mark AE, et al Definition and testing of the GROMOS force-field versions 54A7 and 54B7. Eur Biophys J. 2011;40: 843–856. 10.1007/s00249-011-0700-9 21533652

[51] Darden T, York D, Pedersen L. Particle mesh Ewald: An N⋅log(N) method for Ewald sums in large systems. J Chem Phys. 1993;98.

[52] Hess B, Bekker H, Berendsen HJ, Fraaije JG. LINCS: a linear constraint solver for molecular simulations. J Comput Chem. 1997;18: 1463–1472.

[53] Bussi G, Donadio D, Parrinello M. Canonical sampling through velocity rescaling. J Chem Phys. 2007;126: 014101 10.1063/1.2408420 17212484

[54] Mancini G, D’Annessa I, Coletta A, Sanna N, Chillemi G, Desideri A. Structural and Dynamical Effects Induced by the Anticancer Drug Topotecan on the Human Topoisomerase I–DNA Complex. Hofmann A, editor. PLoS ONE. 2010;5: e10934 10.1371/journal.pone.0010934 20532182 PMC2880615

[55] Shao J, Tanner SW, Thompson N, Cheatham TE. Clustering Molecular Dynamics Trajectories: 1. Characterizing the Performance of Different Clustering Algorithms. J Chem Theory Comput. 2007;3: 2312–2334. 10.1021/ct700119m 26636222

[56] Daura X, van Gunsteren WF, Mark AE. Folding–unfolding thermodynamics of a β-heptapeptide from equilibrium simulations. Proteins Struct Funct Bioinforma. 1999;34: 269–280. 10.1002/(sici)1097-0134(19990215)34:3<269::aid-prot1>3.0.co;2-3 10024015

[57] Chovancova E, Pavelka A, Benes P, Strnad O, Brezovsky J, Kozlikova B, et al CAVER 3.0: A Tool for the Analysis of Transport Pathways in Dynamic Protein Structures. Prlic A, editor. PLoS Comput Biol. 2012;8: e1002708 10.1371/journal.pcbi.1002708 23093919 PMC3475669

[58] Humphrey W, Dalke A, Schulten K. VMD: visual molecular dynamics. J Mol Graph. 1996;14: 33–38, 27–28. 8744570 10.1016/0263-7855(96)00018-5

[59] Pettersen EF, Goddard TD, Huang CC, Couch GS, Greenblatt DM, Meng EC, et al UCSF Chimera—a visualization system for exploratory research and analysis. J Comput Chem. 2004;25: 1605–1612. 10.1002/jcc.20084 15264254

[60] Daidone I, Amadei A. Essential dynamics: foundation and applications. Wiley Interdiscip Rev Comput Mol Sci. 2012;2: 762–770. 10.1002/wcms.1099

[61] Tatoli S, Zazza C, Sanna N, Palma A, Aschi M. The role of Arginine 38 in horseradish peroxidase enzyme revisited: A computational investigation. Biophys Chem. 2009;141: 87–93. 10.1016/j.bpc.2008.12.015 19178992

[62] Shah MB, Wilderman PR, Pascual J, Zhang Q, Stout CD, Halpert JR. Conformational Adaptation of Human Cytochrome P450 2B6 and Rabbit Cytochrome P450 2B4 Revealed upon Binding Multiple Amlodipine Molecules. Biochemistry (Mosc). 2012;51: 7225–7238. 10.1021/bi300894z PMC3485383 22909231

[63] Shahrokh K, Cheatham TE, Yost GS. Conformational dynamics of CYP3A4 demonstrate the important role of Arg212 coupled with the opening of ingress, egress and solvent channels to dehydrogenation of 4-hydroxy-tamoxifen. Biochim Biophys Acta BBA—Gen Subj. 2012;1820: 1605–1617. 10.1016/j.bbagen.2012.05.011 PMC3404218 22677141

[64] LaRonde-LeBlanc N, Wlodawer A. Crystal Structure of A. fulgidus Rio2 Defines a New Family of Serine Protein Kinases. Structure. 2004;12: 1585–1594. 10.1016/j.str.2004.06.016 15341724

