# Supplementary figures and images for: F429 Regulation of Tunnels in Cytochrome P450 2B4: A Top Down Study of Multiple Molecular Dynamics Simulations
Source: PLoS One. 2015 Sep 28;10(9):e0137075. doi: 10.1371/journal.pone.0137075 (PMC4587367; doi:10.1371/journal.pone.0137075)

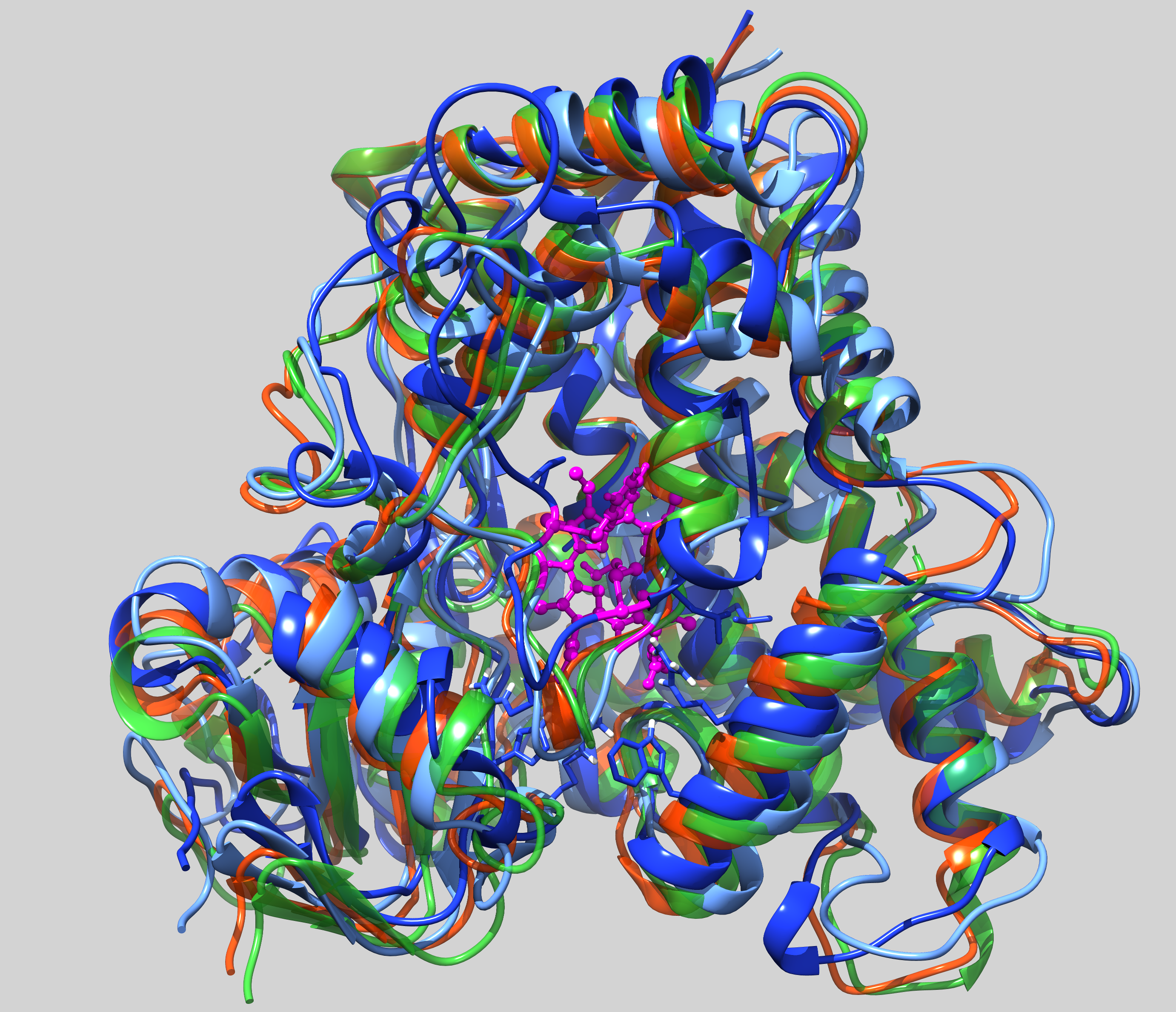

Supplement: S6 Fig — The centroid of WT (blue ribbons), and F429H (cyan ribbons) simulations together with the 1SUO (orange ribbons) and 4MGJ (light green ribbons). (TIFF) [file pone.0137075.s006.tiff]
