# Supplementary material for: F429 Regulation of Tunnels in Cytochrome P450 2B4: A Top Down Study of Multiple Molecular Dynamics Simulations
Source: PLoS One. 2015 Sep 28;10(9):e0137075. doi: 10.1371/journal.pone.0137075 (PMC4587367; doi:10.1371/journal.pone.0137075)
Supplement: S1 Table — The system in which a specific bond was observed, the donor and acceptor atom and the relative stability (with respect to a total of 140 ns) are shown. “heme-A” or “heme-D” stand for heme-priopionate group A or D (see Fig 2). Interactions are shown if the lifetime is grater than 60% of used configurations (i.e. 91 ns of sampling) Atom names N/O without further specification indicate backbone atoms; N* and O* for Arg and Glu or Asp residues indicate either nitrogen or oxygen atom of the side chain; the sum of the interactions is shown. (DOCX) [file pone.0137075.s008.docx]

**Table S1.**

| System | Donor | Acceptor | Lifetime (% frames) |
| --- | --- | --- | --- |
| F429A | R98 - N* | OOH^-^ | 94.6 |
| F429A | S210 - Og | F202 - O | 97.8 |
| F429E | S210 - Og | F202 - O | 98.8 |
| F429H | S210 - Og | F202 - O | 97.9 |
| F429L | S210 - Og | F202 - O | 93.9 |
| F429A | S210 - Og | F206 - O | 94.7 |
| F429E | S210 - Og | F206 - O | 90.3 |
| F429H | S210 - Og | F206 - O | 95.8 |
| F429L | S210 - Og | F206 - O | 91.8 |
| WT | E301 - N | F297 - O | 86.7 |
| F429L | E301 - N | F297 - O | 66.0 |
| F429A | T302 - Og | A298 - O | 66.8 |
| F429E | T302 - Og | A298 - O | 81.3 |
| F429H | T302 - Og | A298 - O | 91.4 |
| F429L | T302 - Og | A298 - O | 81.9 |
| WT | T302 - Og | OOH^-^ | 94.8 |
| WT | R308 - N* | E301 - O* | 62.1 |
| WT | H369 - Ne | heme_A | 63.4 |
| F429A | S430- Og | heme_A | 88.2 |
| F429E | S430- Og | heme_A | 84.7 |
| F429H | S430- Og | heme_A | 75.7 |
| F429A | R434 - N | heme_A | 96.5 |
| F429E | R434 - N | heme_A | 74.8 |
| F429H | R434 - N | heme_A | 94.2 |
| F429L | R434 - N | heme_A | 83.9 |
| F429L | I435 - N | K433 - O | 66.8 |
| F429A | I435 - N | heme_A | 92.9 |
| F429H | R125 – N* | I435 - O | 78.9 |
| F429A | L437 - N | heme_D | 68.3 |
| F429E | L437 - N | heme_D | 85.2 |
| F429L | L437 - N | heme_D | 92.9 |
| WT | I441- N | L437 - O | 81.1 |
| F429L | I441 - N | E439 - O* | 89.4 |
| F429L | T444 – Og | G440 - O | 86.9 |

**Table S1**. **Differences in Hydrogen bond networks in the WT, F429A, F429E, F429H and F429L trajectories.** The system in which a specific bond was observed, the donor and acceptor atom and the relative stability (with respect to a total of 140 ns) are shown. “heme-A” or “heme-D” stand for heme-priopionate group A or D (see Figure 1). Interactions are shown if the lifetime is grater than 60% of used configurations (i.e. 91 ns of sampling) Atom names N/O without further specification indicate backbone atoms; N* and O* for Arg and Glu or Asp residues indicate either nitrogen or oxygen atom of the side chain; the sum of the interactions is shown.
